# MAGIC populations: a next-generation framework for dissecting complex quantitative traits and accelerating molecular breeding in crops

**DOI:** 10.3389/fpls.2026.1867756

**Published:** 2026-06-30

**Authors:** Asad Ullah, Zhijun Tong, Muhammad Kamran, Umaira, Xuejun Chen, Haiming Xu, Bingguang Xiao

**Affiliations:** 1 Key Laboratory of Tobacco Biotechnological Breeding, National Tobacco Genetic Engineering Research Center, Yunnan Academy of Tobacco Agricultural Sciences, Kunming, China; 2 Institute of Crop Science and Bioinformatics, College of Agriculture and Biotechnology, Zhejiang University, Hangzhou, China

**Keywords:** MAGIC populations, QTL mapping, haplotype reconstruction, epistatic interactions, genomic selection, multi-omics integration

## Abstract

Dissecting complex quantitative traits is constrained by limited genetic diversity in biparental populations and population structure confounding in genome-wide association studies. Multi-parent Advanced Generation Inter-Cross (MAGIC) populations address these limitations by intercrossing multiple diverse founders followed by selfing to generate immortalized recombinant inbred lines exhibiting extensive recombination and balanced allele frequencies. MAGIC populations synergistically combine high mapping resolution with broad genetic diversity, enabling detection of small-effect QTLs, epistatic interactions, and genotype-by-environment effects. Despite their immense potential and successful deployment across diverse crops, several critical challenges remain regarding founder selection strategies, computational efficiency of haplotype reconstruction, and seamless integration into existing breeding pipeline. In this review, we synthesize current knowledge of MAGIC construction principles, crossing designs, and inbreeding strategies, and critically evaluate genotyping technologies and statistical frameworks including hidden Markov models, identity-by-descent mapping, and multi-locus mixed models. Furthermore, we explored how integrating with high-throughput phenotyping enhances multi-environment trait characterization, with applications across diverse crops revealing common bottlenecks and successful strategies. We also outlined transformative opportunities through joint linkage-association analysis for causal variant identification, integrating MAGIC Populations with AI-driven genomic selection for accelerated genetic gain, and multi-omics approaches for mechanistic trait dissection. This synthesis provides actionable frameworks for optimizing MAGIC population development and exploitation, advancing precision crop improvement in the face of climate change and resource constraints.

## 1 Introduction

**Figure 1:**
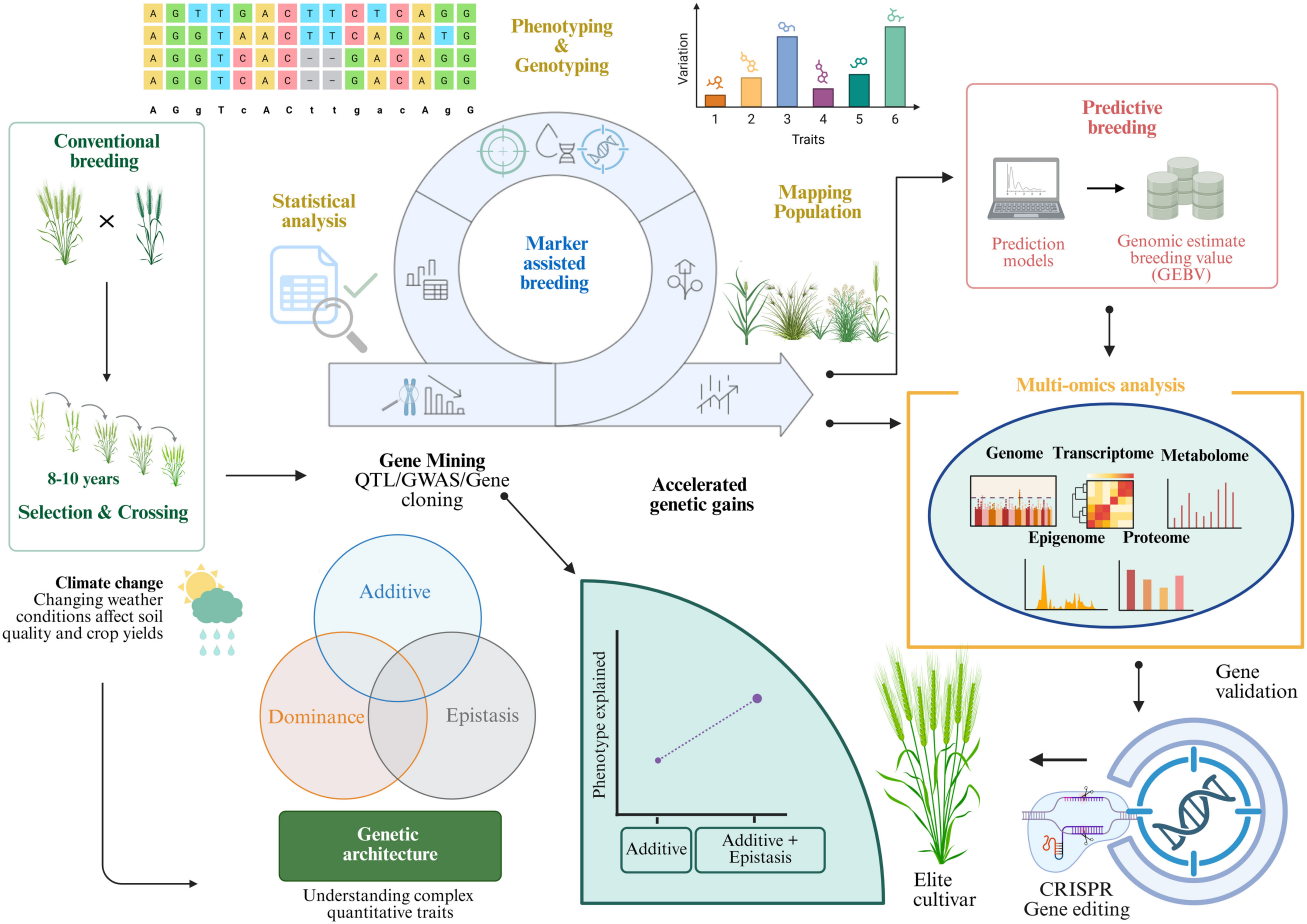
Schematic representation of the transition from time-consuming conventional breeding, challenged by climate change, to efficient marker-assisted breeding. The figure highlights the approaches for dissecting genetic architecture and gene mining through the integration of QTL mapping and GWAS with predictive breeding and multi-omics analyses. This combined strategy enhances trait dissection and candidate gene identification, culminating in gene editing to develop climate-resilient elite cultivars.

Improving crop productivity, resilience, and quality remains a central goal of modern plant breeding, particularly in the face of climate change, emerging diseases, and growing global food demand. Achieving these objectives requires a thorough understanding of the genetic architecture of complex quantitative traits, such as yield, stress tolerance, and disease resistance. These traits are typically controlled by numerous loci with small individual effects and are strongly influenced by genotype-by-environment (*G* × *E*) interactions (Bhat et al., 2016; Vishwakarma et al., 2022; Jeon et al., 2023). Advances in quantitative genetics, molecular biology, and genomics have substantially enhanced our ability to dissect the genetic basis of such traits by characterizing additive, dominance, and epistatic effects, collectively referred to as genetic architecture (Holland, 2007; Almasy and Blangero, 2010). Among these components, additive effects generally account for the largest proportion of heritable phenotypic variation, whereas gene-by-gene (epistatic) and *G* × *E* interactions introduce additional layers of complexity to the genotype-to-phenotype relationship (**Figure 1**).

The molecular genetics revolution, together with advances in high-throughput genotyping and sequencing technologies, has transformed the study of complex traits. Genetic mapping approaches, particularly linkage mapping and genome-wide association studies (GWAS), have become indispensable tools for identifying loci associated with phenotypic variation and accelerating crop improvement (Bernardo, 2020; Wei et al., 2024). Consequently, plant breeding has evolved from traditional phenotype-based selection toward genomics-assisted strategies that integrate genotypic and phenotypic information, thereby improving the precision of trait dissection, gene discovery, and breeding decisions.

Central to the genetic dissection of complex traits is the identification of quantitative trait loci (QTLs) and the causal variants underlying phenotypic variation (Doerge, 2002; Xu et al., 2017). A QTL is defined as a chromosomal region containing one or more genes that contribute to variation in a quantitative trait (Geldermann, 1975). Successful QTL discovery depends on four interconnected components: well-designed mapping populations, dense molecular markers, accurate phenotyping, and robust statistical methods capable of accounting for both genetic and environmental complexity (Doerge, 2002; Collard et al., 2005; Beyer et al., 2008; Arrones et al., 2020). Over the past two decades, linkage mapping and GWAS have emerged as the dominant approaches for trait dissection. However, each has inherent limitations that reduce its effectiveness for resolving the genetic basis of complex traits (Xu et al., 2017).

To overcome the trade-offs between linkage mapping and GWAS, multiparent populations (MPPs) were developed to combine the strengths of both approaches. By integrating multiple founders through successive intercrossing generations, MPPs create highly recombinant populations with broad allelic representation, enabling high-resolution mapping and more accurate identification of loci underlying complex traits (Bohra, 2013; Cockram, James and Mackay, 2018; Li et al., 2021). Several MPP designs have been widely adopted, including nested association mapping (NAM), multi-parent advanced generation intercross (MAGIC), random open-parent association mapping (ROAM), and complete diallel plus unbalanced breeding intercrossing (CUBIC) (Wang et al., 2022; Chen et al., 2025). Among these, MAGIC populations have emerged as one of the most powerful next-generation genetic resources. Through systematic intercrossing of eight or more founder lines, MAGIC populations generate high levels of recombination and genetic diversity, providing superior mapping resolution and broader allelic representation than conventional biparental populations. This review focuses on the principles underlying MAGIC population development, including founder selection, mating designs, and population construction strategies. We compare MAGIC populations with biparental, natural, and other multiparent populations to highlight their relative strengths and limitations for genetic analysis and crop improvement. We further summarize recent advances in genotyping technologies and critically evaluate the statistical frameworks used to analyze the complex haplotype mosaics generated in MAGIC populations, identifying established methodologies, ongoing challenges, and remaining computational bottlenecks. Applications of MAGIC populations across more than 20 crop species are synthesized to reveal recurring patterns in their performance, including the types of genetic variation they effectively capture, their limitations in detecting specific classes of loci, and the factors influencing their utility across different biological contexts. Finally, we discuss emerging opportunities for enhancing the impact of MAGIC populations, including integrated linkage–association mapping for causal variant identification, artificial intelligence-assisted genomic selection, and multi-omics integration for mechanistic trait dissection. By synthesizing recent advances and future perspectives, this review provides a comprehensive resource for researchers and breeders seeking to harness the full potential of MAGIC populations in genomics-enabled plant breeding.

## 2 Overview of experimental populations

### 2.1 Biparental populations

**Figure 2:**
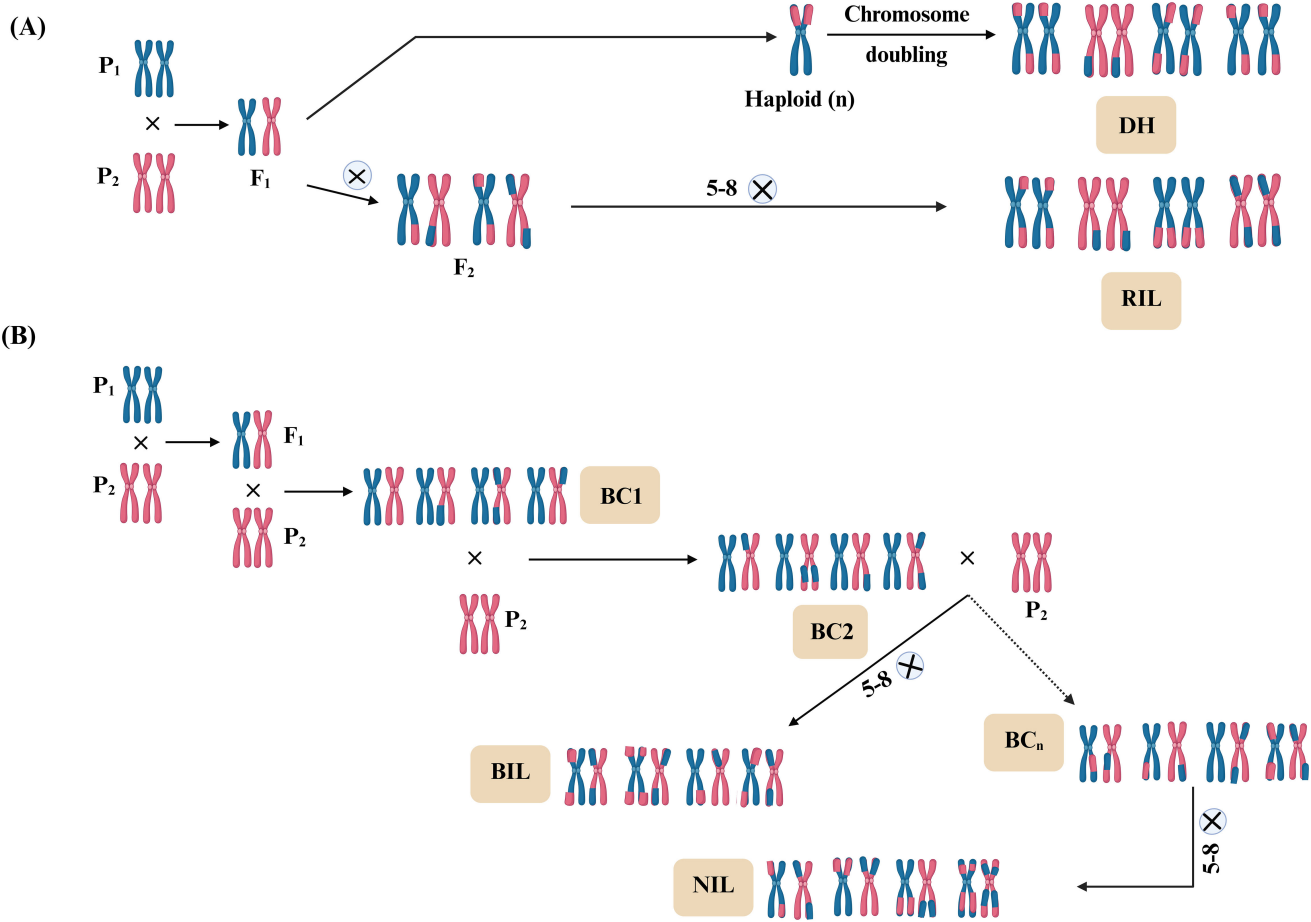
The crossing scheme to develop different bi-parental populations. Panel **(A)** denotes the process of developing the double haploids (DH) through chromosome doubling and the recombinant inbred lines (RIL) through single seed descent method. While panel **(B)** represents the backcross inbred lines (BIL) through repeated backcrossing and the near-isogenic lines (NIL) from single seed descent method.

Biparental mapping populations have long served as foundational tools for QTL discovery due to their simplicity and controlled genetic backgrounds (Xu et al., 2017). These populations are typically developed by crossing two phenotypically contrasting parents to generate F_1_ progeny, followed by selfing, backcrossing, or chromosome doubling to produce segregating populations such as F_2_, backcross (BC), recombinant inbred line (RIL), and doubled haploid (DH) populations (Bohra, 2013; Arrones et al., 2020) (**Figure 2**). More specialized populations, including near-isogenic lines (NILs), chromosome segment substitution lines (CSSLs), and advanced intercross lines (AILs), have further expanded their utility for QTL validation and fine mapping.

Despite their utility, biparental populations suffer from limited genetic diversity and mapping resolution since allelic variation originates from only two parents. This narrow base often leads to broad QTL intervals, containing numerous genes, and reduces mapping resolution (Doerge, 2002; Alqudah et al., 2020; Arrones et al., 2020; Yuan et al., 2023; Zadokar et al., 2025). These limitations are particularly evident in crops with narrow breeding pools or large polyploid genomes, where insufficient polymorphism and extensive linkage blocks can hinder the identification of causal variants. Although increasing population size or using advanced designs such as AILs can partially improve mapping resolution, yet these approaches do not overcome the fundamental constraint of limited founder diversity (Bohra, 2013), thus reducing the effectiveness of these populations for dissecting complex traits (Huang et al., 2012).

#### 2.2 Natural populations

Natural population-based mapping has emerged as a powerful approach for elucidating the inheritance of complex quantitative traits, detecting *G* × *E* interaction, and exploring the genetic basis of heterosis (Wu et al., 2002.; Wu and Zeng, 2001; Widmayer et al., 2022). Natural populations comprise a broad reservoir of genetic variation derived from elite cultivars, landraces, wild relatives, and exotic accessions, collectively capturing extensive allelic richness within a species (Xiao et al., 2012; Xu et al., 2017). Their extensive history of recombination results in reduced linkage disequilibrium (LD) and finer haplotype structure, enabling high-resolution genome-wide association studies (GWAS) and more precise localization of QTLs and candidate genes (Yang et al., 2010; Alqudah et al., 2020). Despite these advantages, the genetic complexity of natural populations presents significant analytical challenges. Population structure, cryptic relatedness, and heterogeneous LD patterns can confound marker–trait associations and increase the likelihood of false-positive signals if not adequately accounted for in statistical models (Wu and Zeng, 2001; Kim et al., 2007). In addition, rare alleles are often poorly represented, limiting the power to detect variants that may contribute substantially to trait variation (Lu et al., 2010). Consequently, while natural populations generally offer superior mapping resolution compared with biparental populations, they often require a trade-off between genetic diversity and analytical complexity. These limitations have motivated the development of multiparent populations, which aim to combine the diversity and recombination advantages of association panels with the controlled genetic structure of designed mapping populations.

### 2.3 Multiparent populations

The MPP concept originated from mouse heterogeneous stocks, in which multiple founders were intermated over generations to intensify recombination and refine QTL localization (Mott et al., 2000). However, high heterozygosity and repeated genotyping restricted their use in plants (Woods and Mott, 2017). To overcome this, stable and reproducible designs such as nested association mapping (NAM), multi-parent advanced generation intercross (MAGIC), and random-open parent association mapping (ROAM) were developed for crops (Wang et al., 2022; Yin et al., 2024) (**Table 1**).

**Table 1:** Comparison of biparental, natural, NAM and MAGIC for genetic mapping.

Characteristic	Biparental	Natural population	NAM	MAGIC
Founders	2	Diverse germplasm	Multiple diverse lines × common parent	Multiple diverse founders (4–16+)
Genetic diversity	Low	High	Moderate–High	High
Allelic variation per locus	Biallelic	Multi-allelic	Mostly biallelic within families	Multi-allelic
Allele frequency balance	Fixed (1:1)	Often skewed; rare alleles common	Balanced within families; biased toward common parent	Designed to be balanced across founders
Recombination events	Limited	Accumulated over evolutionary time	Family-based recombination	Extensive intercross recombination
Mapping resolution	Low–Moderate	Very High	Moderate	High
Statistical power	High for large-effect QTL	Moderate; depends on MAF and structure correction	High for rare and family-specific alleles	High across effect sizes
Population size	Small–Moderate	Large	Large	Moderate–Large
Population structure	Minimal	Strong; requires correction	Family-structured	Controlled but complex
Establishment time	Moderate	None (existing germplasm)	Long	Long
Establishment cost	Moderate	Low	High	High
Genotyping complexity	Low	Moderate	High	High
Computational demand	Low–Moderate	Moderate–High	High	High
Breeding relevance	Limited (narrow base)	Variable	High (elite × diverse design)	High (balanced breeding germplasm)
Main advantages	Simple design; strong effect detection	Highest resolution; broad diversity	Rare allele discovery; structured power	Balanced allele frequencies; high resolution and power
Main limitations	Low resolution; limited diversity	Structure confounding; rare allele issues	Complex design; common parent bias	Time/resource intensive; complex haplotypes

The table summarizes the comparative analysis, synthesizes key genetic, methodological, and practical attributes across the major population types used in quantitative trait dissection.

Among these MPP designs, each imposes its own trade-offs between allelic diversity, population structure, and analytical tractability. Nested association mapping (NAM) links diversity to a single common parent, concentrating statistical power but creating asymmetric parental contributions that bias haplotype representation (Xiao et al., 2016, 2017; Scott et al., 2020). Random-open-parent association mapping (ROAM) avoids a shared ancestor but is prone to unequal allele frequencies that elevate false-positive rates (Chen et al., 2023; Yin et al., 2024). MAGIC populations resolve these limitations through their defining design principle: systematic, multi-generation intercrossing of multiple founders to achieve balanced representation, high recombination, and minimal confounding.

## 3 MAGIC population: concept and construction

### 3.1 Historical context and core concept

Although (Kover et al., 2009) is widely cited as the origin of MAGIC populations in plants, the design was first implemented in upland cotton in 2002 (Islam et al., 2016), a priority that is frequently overlooked. Both implementations share the defining features of the MAGIC framework: systematic multi-generation intercrossing among multiple founders followed by inbreeding to produce immortalized RILs whose genomes are fine-scale mosaics of all parental haplotypes (Samantara et al., 2021). The biological consequences of this design, including reduced linkage disequilibrium, increased recombination breakpoint density, and balanced multi-allelic representation, are well documented and implemented across multiple crops (Stadlmeier et al., 2018; Yuan et al., 2023). However, these properties are not guaranteed by the design alone; they depend critically on decisions made at each construction stage, regarding the selection of the parents, the crossing design, and the inbreeding strategies. The following sections therefore treat MAGIC construction not as a procedural sequence but as a series of trade-off decisions with measurable consequences for analytical power.

### 3.2 Founder selection

**Figure 3:**
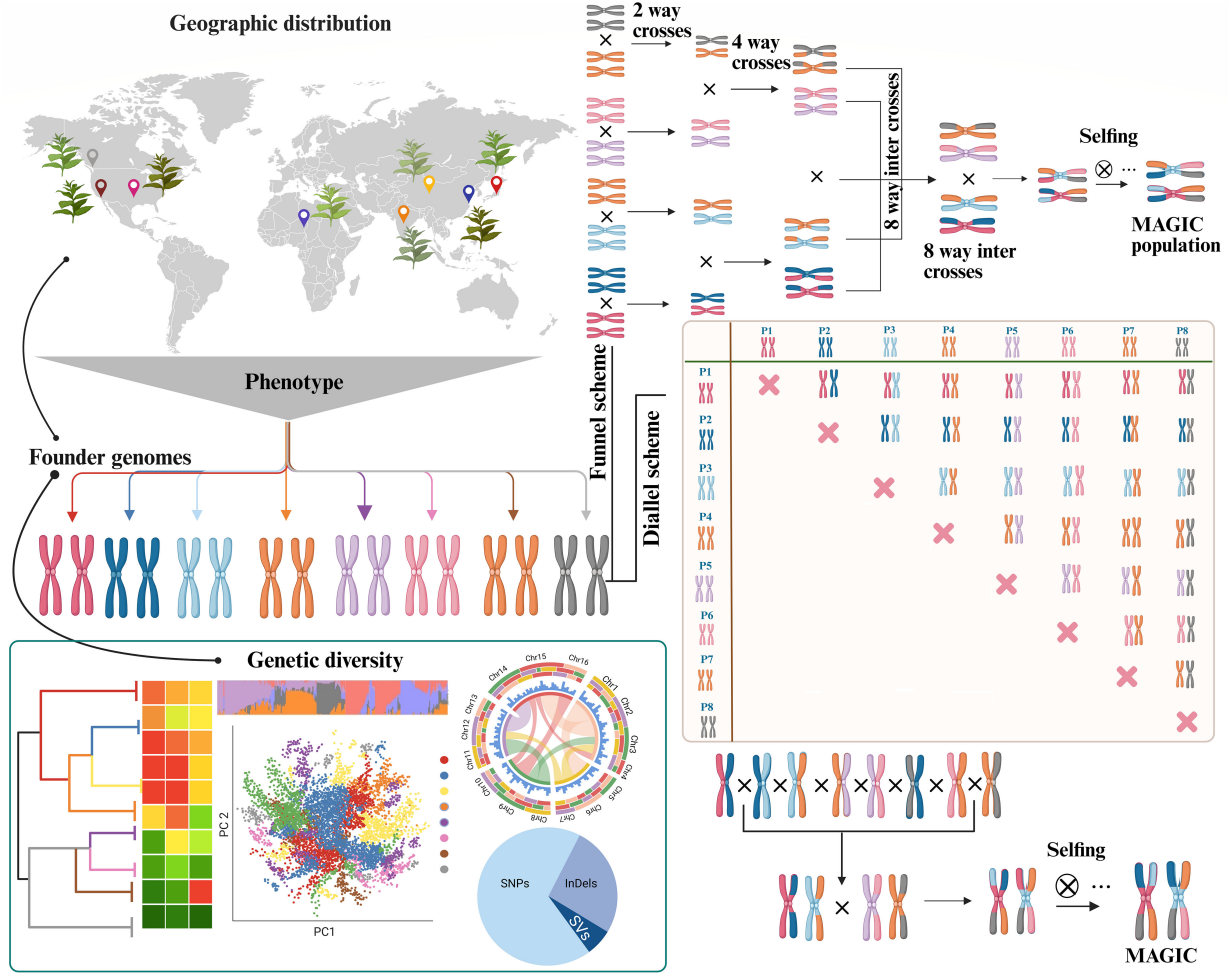
Founder selection strategies based on geographic, phenotypic and genotypic diversity with depicts the schematic representation of crossing scheme including 8 founders for both funnel and diallel designs.

Founder selection is the most critical step in MAGIC population development because the allelic diversity and genetic architecture represented in the final recombinant inbred line (RIL) panel are entirely determined by the founders. The primary challenge lies in balancing genetic diversity with reproductive compatibility. While maximizing genetic diversity increases the likelihood of capturing alleles with meaningful phenotypic effects, excessive divergence among founders can compromise population development through fertility barriers, segregation distortion, and linkage drag (Samantara et al., 2021; Huang et al., 2023). Consequently, MAGIC populations are typically developed using founders drawn from relatively compatible germplasm categories, such as elite cultivars, landraces, or carefully selected exotic accessions, rather than from highly divergent genetic backgrounds. Within these categories, founders are selected to maximize genetic distinctiveness and phenotypic complementarity while maintaining sufficient crossing compatibility. Beyond genetic diversity, phenotypic complementarity among founders is an important consideration because it increases the potential for transgressive segregation and the generation of novel allele combinations (**Figure 3**). Equally important is reproductive compatibility, as successful intercrossing is essential for maintaining population size and ensuring balanced founder representation. Accordingly, founders are typically highly inbred lines with proven fertility and crossing ability. Prior molecular characterization further facilitates haplotype reconstruction and improves the accuracy of downstream quantitative trait locus (QTL) detection.

Recent advances in genomic technologies have enabled a more systematic and data-driven approach to founder selection. Genome-wide marker datasets can be used to assess genetic diversity through distance matrices and kinship estimates, while simulation tools such as the *magicdesign* R package (Ladejobi et al., 2016) allow researchers to evaluate alternative founder combinations and crossing schemes before initiating population development. These approaches represent a substantial improvement over purely empirical or phenotype-based selection, although their effectiveness depends on the availability and quality of genomic resources, which remain variable across species (Sun et al., 2022; Yadav et al., 2025). In polyploid crops, founder selection is further complicated by subgenome-specific variation, as conventional diversity metrics may overlook imbalances among homeologous genomes that can ultimately reduce mapping power and resolution. Therefore, effective founder selection requires a careful balance between maximizing genetic diversity, phenotypic complementarity, and reproductive compatibility to ensure the successful development of a stable and genetically informative MAGIC population (Arrones et al., 2020).

### 3.3 Crossing design

The crossing scheme determines how thoroughly founder genomes are recombined, how equitably each founder contributes to the final population, and how tractable construction is logistically. Two schemes are used: funnel crossing, in which founders are merged hierarchically through sequential two-way, four-way, and higher-order crosses, and diallel crossing, in which all founder pairs are crossed before systematic intercrossing (Samantara et al., 2021. The funnel design minimizes the number of required crosses and is especially useful when founders are difficult to hybridize, such as wild relatives or small-flowered lines (Pascual et al., 2015; Campanelli et al., 2019) (**Figure 3**). However, the sequential crossing order limits opportunities for recombination among all founder genomes in early generations and can lead to unequal founder representation. The diallel design involves crossing all possible founder pairs (half-diallel), followed by systematic intercrossing to combine all genomes within a generation. Although substantially more labor-intensive, this approach produces a broader range of allelic combinations and maintains a more balanced parental contribution (Cockram James and Mackay, 2018). A pioneering example of the complex half-diallel crossing scheme is the upland cotton MAGIC population, in which 11 diverse founders were intercrossed to generate 55 F_1_ half-diallel combinations that were subsequently advanced to produce 547 RILs, successfully integrating a wide array of fiber quality and yield-related alleles (Islam et al., 2016). The choice between funnel and diallel designs, therefore, reflects a trade-off between logistical feasibility and the extent of recombination achievable.

### 3.4 Intercrossing and inbreeding

Following the initial crossing, several rounds of advanced intercrossing are conducted to maximize recombination. Simulation studies recommend at least six intercross generations to optimize genome structure and increase QTL detection power (Kover et al., 2009; Samantara et al., 2021). At this stage, population structure and founder representation are assessed using cluster analysis or principal component analysis (PCA) to verify recombination balance (Wada et al., 2017; Yan et al., 2020). The final step is to generate nearly homozygous lines through either SSD, over five to eight selfing generations, or DH production, which achieves fully homozygosity in one step. For instance, an eight-founder MAGIC population can produce ~1, 000 RILs through SSD, while DH methods, as in barley MAGIC populations, substantially shorten development time (Cockram James and Mackay, 2018). The resulting RILs are immortal resources containing fine-scale genomic mosaics of all founders. Once genotyped, they enable repeated use for high-resolution QTL mapping, candidate gene discovery, and studies of *G* × *E* interactions, providing a powerful platform for both genetic research and breeding (Arrones et al., 2020; Samantara et al., 2021).

## 4 Key advantages of MAGIC populations

The structural characteristics of MAGIC populations provide several advantages over traditional biparental and natural populations for genetic analysis. The extensive recombination accumulated through multiple generations of intercrossing reduces linkage disequilibrium (LD) more effectively than other mapping populations, enabling finer mapping resolution and more precise localization of QTLs (Kover et al., 2009; Ladejobi et al., 2016; Capilla-Pérez et al., 2024). Balanced founder contributions increase the frequency of rare alleles in the population, improving statistical power for detecting small-effect QTLs that biparental studies routinely miss (Puglisi et al., 2021). In addition, MAGIC populations facilitate the evaluation of multiple alleles at a single locus and provide opportunities to investigate epistatic interactions and *G* × *E* effects within a unified framework (Wang et al., 2024).

Because MAGIC populations consist of immortal recombinant inbred lines (RILs), they can be repeatedly phenotyped across multiple environments and years, making them particularly valuable for dissecting complex traits and their environmental responses (Velmurugan et al., 2018). From a breeding perspective, the QTL, haplotype, and genomic prediction information generated from MAGIC populations can be directly incorporated into pre-breeding and cultivar development programs, facilitating the selection of favorable allele combinations that may be inaccessible through conventional elite-by-elite crosses (Scott et al., 2020; Arrones et al., 2020). Nevertheless, the magnitude of these advantages depends on population design and analytical implementation. The multi-allelic architecture that improves the detection of rare variants also increases model complexity and often requires larger population sizes than biparental populations to achieve comparable statistical power for individual loci (Diaz et al., 2020). Likewise, the superior mapping resolution frequently attributed to MAGIC populations is strongly influenced by founder number, population size, recombination history, and marker density, and may not always exceed that achieved in well-powered GWAS panels.

## 5 Limitations and challenges

Despite their considerable value for genetic analysis and crop improvement, MAGIC populations present several practical and analytical challenges. The most immediate limitation is the substantial investment of time and resources required for population development. Multi-generation intercrossing, large-scale population maintenance, and extensive multi-environment phenotyping require long-term commitments that may exceed the capacity of many breeding programs, particularly when parental incompatibility reduces progeny production (Thakur and Deshmukh, 2022; Xiao et al., 2022; Yuan et al., 2023). Furthermore, the costs associated with high-density genotyping and advanced phenotyping platforms can limit the scalability and broader adoption of MAGIC populations (Houle et al., 2010). From an analytical perspective, unequal founder representation caused by differential fertility, selection, or genetic drift can introduce population structure and distort LD patterns, potentially generating spurious marker–trait associations. Although kinship-based linear mixed models help mitigate these effects, their performance depends on accurate haplotype reconstruction and appropriate modeling assumptions (Arrones et al., 2020). When founder contributions become highly unbalanced, some degree of bias and false-positive associations may remain unavoidable.

Polyploid species present additional challenges because homoeologous chromosomes, allele dosage variation, and homeologous exchanges complicate both haplotype reconstruction and founder assignment (Grandont et al., 2014; Deb et al., 2023). To address these issues, researchers increasingly employ subgenome-specific marker systems and ploidy-aware genotype-calling methods. Software tools such as *polyRAD*, *updog*, and EBG incorporate Bayesian frameworks that improve genotype accuracy and support reliable GWAS and genomic prediction analyses in polyploid crops (Njuguna et al., 2023).

In species where doubled haploid (DH) production is feasible, DH technology can accelerate the fixation of recombinant genomes and reduce segregation distortion following the initial intercrossing phase. For vegetatively propagated and perennial crops, including potato, cassava, banana, and sugarcane, which often exhibit severe inbreeding depression, high heterozygosity, and self-incompatibility, the MAGIC concept can be adapted into clonal or pseudo-MAGIC populations maintained through vegetative propagation. For perennial crops with long juvenile phases, speed breeding protocols and marker-assisted generation advancement can help reduce generation intervals and accelerate population development (Flachowsky et al., 2011; Nocker and Gardiner, 2014). Taken together, the effectiveness of MAGIC populations depends on careful population design, comprehensive genomic characterization of founders, and adequate population size (Pascual et al., 2016; Wei and Xu, 2016; Yang et al., 2021).

## 6 Genotyping strategies

### 6.1 Next-Generation Sequencing (NGS)

The resolution of a MAGIC population can ultimately depend on how completely its recombinant mosaics can be genotyped. The recombination accumulated during construction becomes informative only when marker coverage is dense enough to trace each founder haplotype across the genome, so the choice of genotyping platform directly shapes the analytical power of the resource. Traditional marker platforms, including DArT markers, SSRs (Huang et al., 2012), fixed SNP arrays (Cavanagh et al., 2013; Wang et al., 2014; Huang et al., 2015), and Kompetitive Allele Specific PCR (KASP) assays, provide high-throughput and cost-effective genotyping but are limited to predefined variants. This ascertainment bias is particularly problematic in MAGIC populations because these platforms often underrepresent rare and founder-specific alleles, reducing their ability to capture the full spectrum of genetic variation generated through multi-parent intercrossing (Samantara et al., 2021). As a result, only a subset of recombination events can be detected, leading to reduced haplotype resolution and mapping precision (Gardner et al., 2016; Stadlmeier et al., 2018). These limitations are especially pronounced in species with large, polyploid, or poorly characterized genomes, where comprehensive variant catalogs are often unavailable (Bhat et al., 2016).

Next-Generation Sequencing (NGS) addresses these limitations by detecting polymorphisms genome-wide at nucleotide resolution, without prior knowledge of variant sites and largely independent of genome size or ploidy (Bhat et al., 2016; Scott et al., 2020). In MAGIC populations, NGS contributes in two complementary ways. First, it facilitates the generation of high-quality chromosome-scale genome assemblies for founder lines (Mascher et al., 2017; Scott et al., 2020; Samantara et al., 2021). These assemblies can be integrated into pan-genome frameworks that capture both shared and unique genomic content, thereby revealing structural variants and presence–absence variation that may influence gene expression and phenotypic diversity (Golicz et al., 2016; Scott et al., 2020). Second, NGS facilitates high-density genotyping of RILs through WGS or reduced representation sequencing (RRS) methods like RAD-seq and GBS. By capturing a broader spectrum of polymorphism, including both common and rare variants, these approaches provide the dense marker data necessary to dissect the intricate recombination patterns within MAGIC populations.

Although RRS is more cost-effective, it involves complex sample preparation that can introduce bias and missing data. Moreover, targeting less variable regions, such as coding sequences, can yield a lower density of polymorphic markers (Scott et al., 2020; Samantara et al., 2021). Overall, NGS empowers MAGIC research by combining high-resolution founder genome assembly with dense genotyping, facilitating the dissection of complex genetic architecture with unprecedented precision.

### 6.2 Genotyping-by-Sequencing (GBS)

Among NGS-based genotyping platforms, genotyping-by-sequencing (GBS) has emerged as one of the most widely adopted approaches for MAGIC populations because it offers an effective balance between marker density and cost (Bhat et al., 2016). GBS combines marker discovery and genotyping within a single workflow, eliminating the ascertainment bias associated with predefined marker arrays and enabling the detection of population-specific polymorphisms (Bajgain et al., 2016). Additional advantages include simplified library preparation, high multiplexing capacity, and relatively low per-sample costs, making GBS particularly attractive for large mapping populations and species with complex genomes. Consequently, GBS has been used successfully to develop *de novo* genetic maps (Saintenac et al., 2013) and identify QTLs for traits like plant height in barley (Liu et al., 2014b).

However, the cost-efficiency of GBS entails trade-offs, primarily its reduced genome representation, which can cause lower coverage, uneven marker distribution, and high rates of missing data. These issues may affect downstream analyses and therefore require robust bioinformatics pipelines for data imputation and quality control (Scott et al., 2020; Samantara et al., 2021). Nevertheless, ongoing advances in sequencing technologies and computational methods continue to improve the utility of GBS. Lower sequencing costs, improved imputation algorithms, and integration with third-generation sequencing (TGS) platforms are enhancing SNP discovery, haplotype phasing, and recombination breakpoint identification. The long, high-fidelity reads generated by modern TGS technologies further improve founder haplotype reconstruction and facilitate more accurate mapping of complex genomic regions (Bhat et al., 2016; Delaneau et al., 2013). These innovations collectively strengthen parental haplotype resolution and increase mapping power and precision for dissecting complex traits and advancing crop breeding.

## 7 Phenotyping strategies

Rapid advances in genotyping have outpaced phenotyping capacity, creating a major bottleneck in the genetic dissection of complex traits (Song et al., 2021). The utility of MAGIC populations depends on accurate and large-scale phenotyping to capture the full spectrum of phenotypic variation. Traditional manual phenotyping is often too labor-intensive, time-consuming, and error-prone to characterize the hundreds to thousands of recombinant inbred lines (RILs) that typically comprise a MAGIC population across multiple environments and trait categories. Consequently, high-throughput phenotyping (HTP) has become a practical necessity rather than a technological convenience for fully exploiting the potential of MAGIC populations (Samantara et al., 2021; Susmitha et al., 2023; Awada et al., 2024).

Modern HTP platforms employ a diverse range of sensing technologies, including RGB imaging, hyperspectral and multispectral cameras, thermal infrared sensors, chlorophyll fluorescence imaging, LiDAR, and UAV-based remote sensing. These technologies enable non-destructive and repeatable measurement of morphological, physiological, and stress-related traits at spatial and temporal scales that are unattainable through conventional approaches (Montes et al., 2007; Furbank and Tester, 2011; Araus and Cairns, 2014; Yang et al., 2020; Awada et al., 2024). Their utility has been demonstrated across numerous crop species, where HTP has facilitated the characterization of complex traits such as canopy architecture, biomass accumulation, photosynthetic efficiency, disease resistance, and stress responses. Moreover, HTP-derived phenotypes have been successfully integrated into GWAS and genomic selection frameworks, improving the detection of trait-associated loci and enhancing predictive breeding accuracy (Rutkoski et al., 2016; Wu et al., 2019). A recurring theme across these applications is that HTP provides the greatest benefit for traits that are difficult to measure manually at scale, exhibit strong temporal dynamics, or require fine spatial resolution.

The specific advantage of HTP for MAGIC populations lies in the ability to repeatedly phenotype stable, immortalized RIL panels across environments and years using standardized protocols. This enables a more comprehensive dissection of *G* × *E* interactions than is possible in transient segregating populations. Furthermore, repeated measurements throughout plant development facilitate the analysis of trait trajectories rather than single time-point observations, creating opportunities to identify dynamic QTLs and developmental stage-specific genetic effects that are often overlooked in conventional studies (Huang et al., 2015; Samantara et al., 2021). Recent advances in HTP have also contributed to the emergence of phenomics, an interdisciplinary field that integrates biology, bioinformatics, statistics, engineering, and artificial intelligence (AI), further expanding the analytical potential of MAGIC populations. Nevertheless, significant challenges remain. The high-dimensional and temporally structured nature of HTP data requires advanced computational and statistical frameworks capable of jointly modeling genomic, environmental, and phenotypic variation. Although substantial progress has been made, many of these analytical approaches remain underdeveloped and have yet to be systematically evaluated across crop species and MAGIC population designs (Houle et al., 2010; Awada et al., 2024).

## 8 Genetic and phenotypic analysis of MAGIC populations

### 8.1 Phenotypic analysis

Phenotypic analysis of MAGIC populations follows similar principles as biparental designs, but must account for their multiple founder alleles and greater genetic complexity. Mixed linear models (MLM) are typically employed to partition phenotypic variance into genetic, environmental, and genotype-by-environment (G×E) components, forming the basis for heritability estimation and trait selection. MLMs generate best linear unbiased predictions (BLUPs) and best linear unbiased estimates (BLUEs), which adjust raw phenotypic data for environmental and experimental effects, thereby increasing the accuracy and power of genetic association analyses in GWAS and linkage mapping. These models can also estimate genetic correlations among traits by fitting multiple-trait models that include both genetic and residual covariances. Several R packages like lme4 (Bates et al., 2015; Jiang et al., 2020) and sommer (Covarrubias-Pazaran, 2023), facilitate MLM-based variance component estimation. In addition, phenotypic correlations between paired traits are commonly assessed using the Pearson correlation coefficient. These correlation analyses assist breeders in identifying traits that can be selected independently (i.e., traits with low correlations to others) and those that require joint selection (i.e., traits exhibiting strong positive or negative correlations with other traits).

### 8.2 Genetics and statistical analysis

The genetic complexity of MAGIC populations necessitates robust analytical frameworks for accurate trait dissection. While conventional single-marker tests (*t*-test, ANOVA, simple linear regression) efficiently identify individual marker-trait associations, they lack genomic context and fail to account for marker ordering and linkage relationships essential for comprehensive QTL detection. Genetic map construction addresses these limitations by establishing marker order and chromosomal positions through recombination frequency estimation, with linked markers assigned to linkage groups based on LOD scores (Sourdille et al., 1996; Timmerman-Vaughan et al., 1996; Varshney et al., 2000; Doerge, 2002; Cockram James and Mackay, 2018). The accumulated recombination events across multiple generations in MAGIC populations create fine-scale founder haplotype mosaics, requiring specialized analytical approaches. Huang et al. (2012) established a comprehensive pipeline for MAGIC analysis encompassing: (1) linkage map construction, (2) haplotype mosaic reconstruction, (3) multi-parent QTL mapping, and (4) LD-based association mapping.

#### 8.2.1 Linkage map construction

Linkage map construction in MAGIC populations requires statistical frameworks capable of modeling multiple founders, complex pedigrees, and numerous recombination breakpoints. Traditional mapping methods developed for biparental populations are generally unable to accommodate these features, necessitating the development of specialized approaches for multi-parent populations. The suitability of commonly used software packages for biparental, cross-pollinated, and multi-parent designs is summarized in **Table 2**. Following high-density genotyping of founder lines and recombinant inbred lines (RILs), markers undergo rigorous quality control to remove loci with excessive missing data, segregation distortion, or redundancy. Co-segregating markers are subsequently grouped into bins, similarity matrices are constructed, and iterative optimization algorithms are applied to determine marker order and estimate recombination fractions (Doerge, 2002).

**Table 2:** List of Software used for linkage map construction.

Software	BP	CP	MP	Reference
JoinMap	✓✓	✓	✓	(Van and Wageningen, 2019)
MapMaker	✓✓	×	×	(Lander et al., 1987)
RECORD	✓✓	×	✓	(Van Os et al., 2005)
CarthaGene	✓✓	×	×	(Schiex and Gaspin, 1997)
AntMap	×	✓✓	×	(Iwata and Ninomiya, 2006)
MSTMAP	✓✓	×	✓	(Mohseni and Lonardi, 2025)
MadMapper	✓✓	×	×	(Kozik and Michelmore, 2006)
Lep-MAP	✓✓	✓✓	✓✓	(Rastas et al., 2013)
CRI-MAP	✓✓	✓✓	×	(Coxmatisea et al., 1995)
THREaD Mapper	✓✓	✓	×	(Cheema et al., 2010)
SeSAM	✓✓	✓	×	(Vidal et al., 2022)
OneMap	✓✓	✓✓	✓✓	(Margarido et al., 2007)
HetMapps	✓	✓✓	×	(Hyma et al., 2015)
MapDisto	✓✓	✓✓	×	(Lorieux, 2012)
FsLinkageMap	✓	✓✓	✓	(Tong et al., 2010)
magicMap	✓✓	✓	✓✓	(Zheng et al., 2019)
HighMap	✓	✓✓	×	(Liu et al., 2014a)
R/qtl	✓✓	×	✓	(Broman et al., 2003)
R/mpmap	✓	✓	✓✓	(Huang and George, 2011)

Most commonly used software for linkage map construction in experimental population, BP denotes bi-parental populations, CP for cross-pollinated populations, and MP denotes multi-parental populations. While ✓✓ represents that specifically designed for that population, ✓ not specifically designed but support in that population and × represents that specific software was not suitable for that population.

The limitations of conventional mapping tools led to the development of MAGIC-specific software based largely on Hidden Markov Models (HMMs), which trace founder haplotype inheritance through probabilistic modeling of recombination events. Early HMM-based implementations, including MapMAKER (Lander et al., 1987), CarthaGène (Schiex and Gaspin, 1997), and R/qtl (Broman et al., 2003), established the foundation for modern multi-parent linkage analysis. More recent tools, such as R/mpMap (Huang and George, 2011), R/qtl2 (Broman et al., 2019), R/happy (Mott et al., 2000), magicMap (Zheng et al., 2019), RABBIT (Zheng et al., 2015), and GAPL (Zhang et al., 2019), have extended these approaches by integrating HMMs with likelihood- and Bayesian-based frameworks to accommodate complex multi-founder inheritance patterns.

Despite their methodological differences, these tools largely reflect a common trade-off between haplotype reconstruction accuracy and computational scalability. At one end of the spectrum, RABBIT provides highly accurate haplotype reconstruction through Bayesian inference but incurs substantial computational costs that increase rapidly with founder number and pedigree complexity (Zheng et al., 2015). Similar scalability challenges affect HMM-based tools such as MapMAKER, CarthaGène, and R/qtl2, where the state space expands dramatically as population complexity increases (Broman et al., 2019). Other software packages improve computational efficiency but introduce functional constraints. For example, R/happy focuses on founder haplotype inference without providing full linkage-mapping functionality (Arrones et al., 2020), whereas R/mpMap is largely restricted to funnel-based MAGIC designs and has limited flexibility for handling missing founder genotypes or non-standard population structures (Huang and George, 2011). Intermediate approaches such as magicMap improve robustness to missing and erroneous genotype calls through multilocus likelihood optimization (Zheng et al., 2019), while GAPL offers a user-friendly framework for four- and eight-parent populations (Zhang et al., 2019). Consequently, no single software package currently achieves an ideal balance among reconstruction accuracy, computational efficiency, and design flexibility. Nevertheless, these methods have proven effective in practice and have supported the construction of MAGIC linkage maps in numerous crops, including wheat, barley, maize, soybean, common bean, and rice (Huang et al., 2012; Dell’Acqua et al., 2015; Diaz et al., 2020).

Beyond computational considerations, MAGIC linkage mapping also faces fundamental challenges. Unlike biparental populations, recombination events cannot always be directly inferred from biallelic markers because multiple founders may share identical alleles at a given locus. Consequently, recombination fraction estimation becomes increasingly difficult as marker density and pedigree complexity increase. Mapping accuracy therefore depends heavily on the degree of allelic differentiation among founders, and ambiguously inferred recombination events often require imputation or exclusion from subsequent analyses (Ahfock et al., 2014). This challenge is further exacerbated in high-density datasets, where marker-grouping approaches based on fixed LOD-score or recombination-fraction thresholds, such as MSTmap and Lep-MAP3, remain sensitive to missing data and genotyping errors (Rastas et al., 2013; Mohseni and Lonardi, 2025). Future progress is likely to depend on the development of software capable of directly analyzing multi-allelic and haplotype-based markers, robustly handling missing genotypes, and integrating genetic and physical maps to improve marker ordering and recombination inference.

#### 8.2.2 Haplotype mosaic reconstruction

Accurate haplotype mosaic reconstruction is the analytical foundation of MAGIC population analysis, because all downstream QTL mapping depends on correctly assigning each genomic segment in a RIL to its founder of origin. The process uses hidden Markov models (HMMs) in which hidden states represent founder origins and observed states correspond to marker genotypes. By evaluating markers sequentially across the genome, HMMs identify transitions between ancestral haplotypes and estimate identity-by-descent (IBD) probabilities along each chromosome (Huang and George, 2011; Huang et al., 2015). Available software tools differ in the extent to which pedigree information is incorporated. Programs such as R/qtl, mpMap, and RABBIT utilize pedigree-based frameworks that exploit crossing histories to improve founder assignment (Scott et al., 2020; Wang et al., 2022), whereas HAPPY employs a pedigree-free approach that offers greater flexibility but may provide lower reconstruction accuracy when reliable pedigree information is available (Mott et al., 2000; Broman, 2005). The practical choice among these methods therefore involves trade-offs among reconstruction accuracy, computational efficiency, tolerance to missing data, and founder number capacity, as summarized in **Table 3**.

**Table 3:** Comparison of haplotype mosaic reconstruction software for MAGIC populations.

Tool	Model/core mechanism	Pedigree requirement	Computational efficiency	Runtime	Memory (GB)	Error & missing data robustness	Max founder capacity	Practical application
R/qtl2	HMM optimized for high-dimensional data	Required	High	Fast	Moderate	Good	16+	Diversity Outbred, MAGIC, and standard experimental crosses
RABBIT	Fast IBD clustering	Required	High	Moderate	High	Very Robust	16+	Highest precision for complex/custom MAGIC pedigrees
mpMap2	Pedigree-aware HMM	Required	Moderate	Moderate	Moderate	Good	16+	Haplotype reconstruction and genetic map construction
magicMap	Maximum Likelihood + Pedigree	Required	Moderate	Slow	Moderate	Moderate	~8 Founders	Map refinement and recombination hotspot detection
HAPPY	Ancestral haplotype mosaic model	Not required	Low	Moderate	Moderate	Good	8 Founders	Legacy studies; Heterogeneous Stock mice

HMM, Hidden Markov Model; IBD, identity-by-descent; GB, gigabytes.

Marker density is a primary determinant of breakpoint resolution because sparse marker coverage leaves many recombination events undetected, resulting in artificially large haplotype blocks (Running and Faris, 2024), (**Supplementary Figure 1**). This limitation is particularly pronounced in regions with elevated recombination rates, where dense marker coverage is required to accurately localize founder transitions. Conversely, recombination-suppressed regions such as pericentromeric and heterochromatic domains tend to retain large founder blocks regardless of marker density, thereby imposing an inherent limit on mapping resolution (Lou et al., 2013; Bhakta et al., 2015). Population generation also influences reconstruction accuracy. Early-generation populations typically contain extensive heterozygosity and relatively few recombination events, leading to uncertain founder assignments and poorly defined haplotype boundaries. As inbreeding progresses, haplotype blocks become smaller and increasingly fixed, improving IBD inference and founder discrimination. Consequently, many MAGIC populations are advanced to approximately F_6_–F_8_ generations, where residual heterozygosity is substantially reduced while additional generations contribute relatively little additional recombination. Founder number and genotype quality introduce further complexity. Increasing the number of founders from four to eight or more substantially expands the number of possible IBD states, thereby increasing the marker density and informativeness required for reliable founder assignment. Missing genotype calls and genotyping errors further complicate reconstruction by generating false breakpoints or ambiguous haplotype transitions. As a result, the choice of genotyping strategy must be carefully matched to both founder complexity and species-specific genome characteristics.

These challenges are further amplified in allopolyploid crops. Sequence similarity between homeologous chromosomes makes subgenome-specific SNP assignment unreliable with standard alignment pipelines, ambiguous marker placement propagates into misclassification of founder origins, and structural variation between founders such as inversions and presence-absence variants disrupt the recombination patterns that HMMs assume. Homeologous exchanges further blur the distinction between homologous and homeologous recombination in ways that diploid-derived models cannot accommodate. Consequently, haplotype reconstruction in allopolyploids requires sub-genome-aware genotyping strategies, high-quality reference assemblies, and frameworks capable of accommodating severe genomic complexity.

#### 8.2.3 QTL mapping methods

QTL mapping methodologies have evolved from basic Interval Mapping (IM) to sophisticated multi-locus mixed-model frameworks, enabling full exploitation of MAGIC population genetic complexity (Samantara et al., 2021). IM revolutionized trait mapping by scanning marker-flanked genomic regions for trait associations, but proved inadequate for multi-founder populations, lacking power for detecting multiple linked loci (Mott et al., 2000; Lander and Botstein, 1989; Doerge, 2002; Huang et al., 2015). The HAPPY package addressed this limitation by incorporating HMM-derived founder probabilities, successfully applied to the first plant multiparent RILs (Kover et al., 2009; Huang et al., 2015). Composite Interval Mapping (CIM) enhanced IM by incorporating marker cofactors as covariates to control genetic background noise, improving QTL detection accuracy (Zeng, 1994; Kao et al., 1999). For MPPs, CIM was adapted using founder probabilities, implemented in MCQTL (Jourjon et al., 2005) and R/mpMap (Huang and George, 2011), though it retained single-locus scanning limitations. Inclusive Composite Interval Mapping (ICIM) refined CIM through stepwise regression for cofactor selection and incorporated two-dimensional scanning for detecting additive and digenic epistatic interactions. Implemented in QTL IciMapping (Meng et al., 2015) and GAPL (Zhang et al., 2019), ICIM successfully identified QTLs in NAM (Li et al., 2011) and MAGIC populations (Zhang et al., 2017). However, ICIM’s inability to model joint effects of multiple loci limited detection of small-effect and linked QTLs, prompting development of Multiple QTL Mapping (MQM), which simultaneously scans multiple loci within a likelihood framework to increase detection power and mapping precision. Mixed Composite Interval Mapping (MCIM) further integrated CIM within a linear mixed model (LMM) framework, modeling major QTLs as fixed effects and polygenic background as random effects, substantially improving epistatic interaction detection (Zhu, 1998). This approach is implemented in QTLNetwork (Yang et al., 2008), which, although designed for bi-parental populations, can be extended to MPPs.

Linear mixed models, initially developed for GWAS to correct population structure and relatedness, have been widely adopted for MPP QTL mapping. By modeling major QTL effects as fixed and polygenic background as random, LMMs separate signal from background more effectively than cofactor-based approaches, particularly in populations with residual structure from unequal founder contributions (Gatti et al., 2014). Complementing classical QTL mapping, genome-wide association study (GWAS) tools like TASSEL, GAPIT, and PLINK employing single-locus and multi-locus mixed-model methods. TASSEL supports general linear models (GLM) and mixed linear models (MLM), GAPIT extends to multi-locus models (MLMM, FarmCPU), while PLINK specializes in single-locus association testing with population structure correction also with various genotypic and allelic tests. LMMs was first time used in wheat MAGIC population by extending the HAPPY model (Huang and George, 2011) and the results demonstrated superior accuracy in identifying known genes compared to biparental populations (Huang et al., 2012), leading to development of single-locus and multi-locus models for MAGIC (Verbyla et al., 2014b) and NAM populations (Paccapelo et al., 2022). For MAGIC populations specifically, single-locus LMMs model marker effects as random effects while distinctly separating them from polygenic background (Wei and Xu, 2016), implemented in MagicQTL and successfully applied in maize ROAM (Xiao et al., 2016) and soybean NAM (Xavier et al., 2018). Multi-locus LMM models incorporating founder probabilities have been developed for dissecting polygenic trait architecture (Li et al., 2023a). IBD-based GWAS approaches have emerged to fully exploit MAGIC genetic potential. Li et al., 2021 developed an IBD-based multi-QTL mixed model using iterative genome scanning within R package statgenMPP, While, enhancing multi-locus detection, this approach still faced challenges for identifying small-effect QTLs associated with highly polygenic traits. To address this, Li et al., 2023a developed mppQTL, combining genome-wide scanning with group-lasso selection and expectation-maximization empirical Bayes (EMEB) algorithms, robustly detecting small-effect and interacting QTLs.

**Figure 4:**
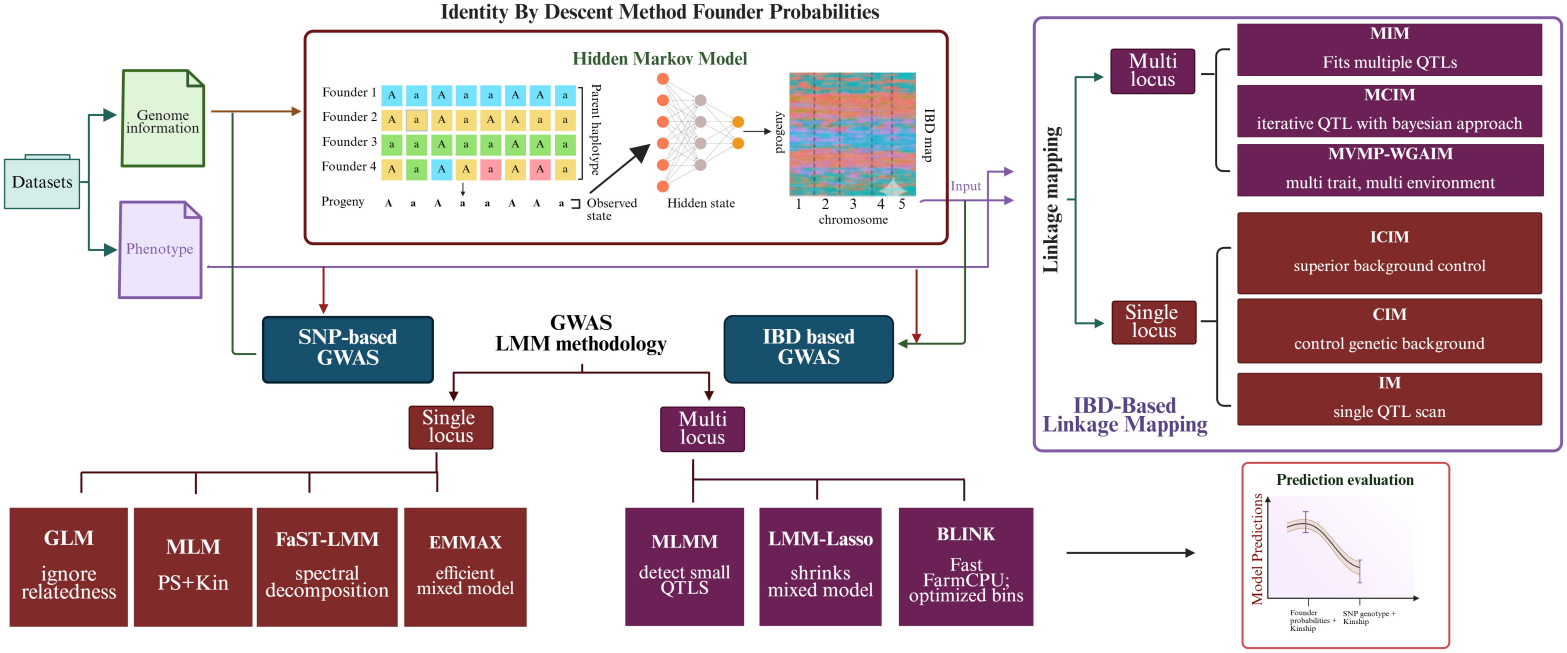
Different statistical models employed for linkage and association mapping to detect and identify QTLs in a MAGIC population, involving the estimation of founder probabilities through HMM.

The LMM framework has been extended to multivariate contexts through Multivariate Multiparent Whole-Genome Average Interval Mapping (MVMP-WGAIM), which simultaneously incorporates all genotype information for multi-trait or multi-environment QTL analysis (Verbyla et al., 2014a; Arrones et al., 2020), This multivariate extension provides deeper insights into the genetic basis of trait–trait and trait–environment relationships. Notably, it has been successfully applied in the wheat MAGIC population developed by (Rebetzke et al., 2014). This evolution from single-locus IM to sophisticated multi-locus LMM frameworks reflects continuous efforts to enhance statistical power and mapping resolution by leveraging MAGIC population genetic richness. The progression of single-locus and multi-locus models for both linkage and association mapping methods is visually depicted in **Figure 4**, with software incorporating these statistical approaches for QTL discovery across all population types summarized in **Supplementary Table 1**. Advanced software packages incorporating these statistical approaches, which include QTL genome scans using Haley-Knott regression, linear mixed models for population and kinship analysis, and the estimation of gene-by-gene and gene-by-environment interactions, are best suited for comprehensive genotype-phenotype interaction studies (Arrones et al., 2020).

## 9 Applications of MAGIC populations in crop improvement

The deployment of Multi-parent Advanced Generation Inter-Cross (MAGIC) populations has evolved from initial proof-of-concept studies in model species such as *Arabidopsis thaliana* to a widely adopted framework for dissecting complex traits across cereals, legumes, horticultural, and industrial crops. The current status of MAGIC populations across crop species is summarized in **Supplementary Table 2**.

### 9.1 Cereals

MAGIC populations have demonstrated broad utility in cereal crops, which form the foundation of global food security. In rice, MAGIC populations have facilitated the dissection of climate-resilience traits, including salinity, submergence, drought, and heavy metal tolerance (Bandillo et al., 2013; Meng et al., 2017; Bossa-Castro et al., 2018; Ogawa et al., 2018; Krishnamurthy et al., 2022; Marrano and Moyers, 2022), as well as nutritional quality traits such as zinc accumulation and magnesium translocation (Meng et al., 2016; Descalsota et al., 2018; Zaw et al., 2019; Ayaad et al., 2021; Liu et al., 2021; Zhi et al., 2023). Despite these successes, rice MAGIC populations that combine divergent indica and japonica founders often exhibit substantial population structure, leading to uneven recombination landscapes and elevated rates of missing data that can complicate haplotype reconstruction and QTL detection. In wheat, the first MAGIC population was developed using four founders (Huang et al., 2012), followed by eight-founder and sixteen-founder populations (Mackay et al., 2014; Scott et al., 2021). These resources have been widely used to map disease resistance loci for powdery mildew, leaf rust, and glume blotch (Gardner et al., 2016; Stadlmeier et al., 2018; Lin et al., 2020; Rollar et al., 2021), while also dissecting complex yield-related traits such as plant height, flowering time, and heading date (Rebetzke et al., 2014; Delhaize et al., 2015; Camargo et al., 2016, 2018; Milner et al., 2016; Sannemann et al., 2018; Fourquet et al., 2024; Shah et al., 2025). The large population sizes typically employed in wheat MAGIC studies have also stimulated the development of specialized analytical methods for linkage map construction, haplotype reconstruction, and marker–trait association analysis. Similarly, barley MAGIC populations have demonstrated enhanced power for detecting both additive and epistatic genetic effects compared with conventional biparental populations, while integration with genomic prediction approaches has improved prediction accuracy for grain yield and related traits (Mathew et al., 2018; Puglisi et al., 2021). However, the extensive recombination accumulated in large-genome cereals can reduce the detectability of minor-effect alleles unless sufficiently large populations are employed. In addition, barley MAGIC populations developed from founders adapted to contrasting climatic conditions have further expanded opportunities for dissecting environmental adaptation and stress resilience (Bülow et al., 2019). MAGIC populations have also been successfully applied in maize and sorghum. In maize, they have enabled the identification of loci controlling senescence, disease resistance, and yield-related traits (Butrón et al., 2018; Mahan et al., 2018; Jiménez-Galindo et al., 2019; Caicedo et al., 2021; Odell et al., 2022), while transcriptome-integrated analyses have provided insights into the regulatory architecture of yield-associated traits (Dell’Acqua et al., 2015). In sorghum, both four-parent and nineteen-parent MAGIC populations have been used to identify candidate genes underlying agronomic performance and drought tolerance (Ongom and Ejeta, 2018; Kumar et al., 2023a, 2023b). A recurring challenge in outcrossing cereals is maintaining genetic purity throughout multiple generations of intercrossing, as pollen contamination can introduce subtle allele-frequency distortions that are difficult to detect without dense genotyping.

### 9.2 Legumes

In legume crops, where domestication bottlenecks have substantially reduced genetic diversity, MAGIC populations provide an effective means of reintroducing favorable alleles from diverse germplasm. In chickpea, eight-parent MAGIC populations have been used to dissect flowering time and drought-related root architecture traits while generating transgressive segregants not accessible through elite × elite crosses (Samineni et al., 2021; Akinlade et al., 2025). Common bean MAGIC populations have identified loci associated with white mold resistance, a trait whose complex genetic architecture often limits progress in biparental populations (Diaz et al., 2020; Escobar et al., 2022). Similarly, the SoyMAGIC panel generated substantial transgressive variation for seed protein and fatty acid composition without the linkage drag commonly associated with wild introgressions (Hashemi et al., 2022). In cowpea and faba bean, MAGIC populations have been used to investigate biotic and abiotic stress resistance, seed quality, agronomic performance, and frost tolerance (Sallam and Martsch, 2015; Khazaei et al., 2018; Olatoye et al., 2019; Ravelombola et al., 2021; Huynh et al., 2024). A persistent limitation across many legume studies is the limited reporting of the phenotypic variance explained by detected QTLs, making cross-study comparisons difficult.

### 9.3 Horticultural crops

Horticultural crops present additional challenges due to high heterozygosity, self-incompatibility, and polyploid genome structures. In tomato, MAGIC populations have successfully combined elite germplasm with wild relative introgressions to identify loci controlling fruit weight and leaf morphology (Pascual et al., 2015; Campanelli et al., 2019; Arrones et al., 2024). In lettuce, the integration of MAGIC populations with high-throughput phenotyping and tissue-specific transcriptomics has facilitated the identification of candidate regulatory loci underlying environmentally sensitive agronomic traits (Chen et al., 2025). In octoploid strawberry, a six-founder MAGIC population has provided a stable mapping resource for tracking multi-allelic variation within a highly complex genome (Wada et al., 2017). Nevertheless, the lack of robust bioinformatic pipelines specifically designed for highly polyploid populations remains a significant analytical challenge.

### 9.4 Industrial crops

In industrial crops, MAGIC populations have proven particularly valuable for improving multiple correlated quality traits simultaneously. In cotton, multi-parent populations have been extensively used to dissect the genetic basis of fiber quality, yield, and disease resistance. Chinese and U.S. cotton MAGIC populations have collectively identified numerous loci associated with fiber length, strength, uniformity, yield performance, and resistance to Fusarium and Verticillium wilts through GWAS, haplotype-based analyses, transcriptomics, and whole-genome sequencing approaches (Islam et al., 2016; Li et al., 2016; Huang et al., 2018, 2021; Naoumkina et al., 2019; Thyssen et al., 2019; Wang et al., 2022; Zhu et al., 2022; Li et al., 2023b; Fang et al., 2023; Mohammed et al., 2024; Ayyaz et al., 2025). These studies highlight the capacity of MAGIC populations to exploit both intraspecific and interspecific diversity for quantitative trait improvement, although fertility barriers and complex chromosome interactions remain obstacles in interspecific designs.

Similarly, in tobacco, where allopolyploid genome structure often complicates the detection of minor-effect loci, an eight-parent MAGIC population enabled the fine mapping of the major nicotine-content locus *qNIC7–1* and identified *NtERF* as its causal regulator (Liu et al., 2022; Yuan et al., 2023). The same population further facilitated the dissection of developmental plasticity in leaf-related traits across multiple environments, identifying loci controlling leaf expansion and uncovering a major structural deletion affecting leaf development (Liu et al., 2024). In Brassica oilseed crops, MAGIC populations have been used to identify key regulators of glucosinolate composition, seed oil content, and disease resistance, demonstrating the ability of multi-parent populations to simultaneously accumulate favorable alleles across correlated quality traits (Fu-yong et al., 2017; Yan et al., 2020). More recently, MAGIC populations in sunflower and jute have revealed substantial transgressive segregation for industrially important traits, including Verticillium wilt resistance and bast fiber yield (Satya et al., 2023; Domínguez et al., 2025). Across all of these industrial crop applications, a major operational challenge remains the high genotype-by-environment interaction, which frequently causes the phenotypic effects of minor-effect QTLs discovered in multi-parent populations to vary significantly across different production environments. Collectively, these studies highlight the versatility of MAGIC populations as a powerful platform for dissecting complex traits and accelerating crop improvement across diverse species and breeding systems.

## 10 Future perspectives

**Figure 5:**
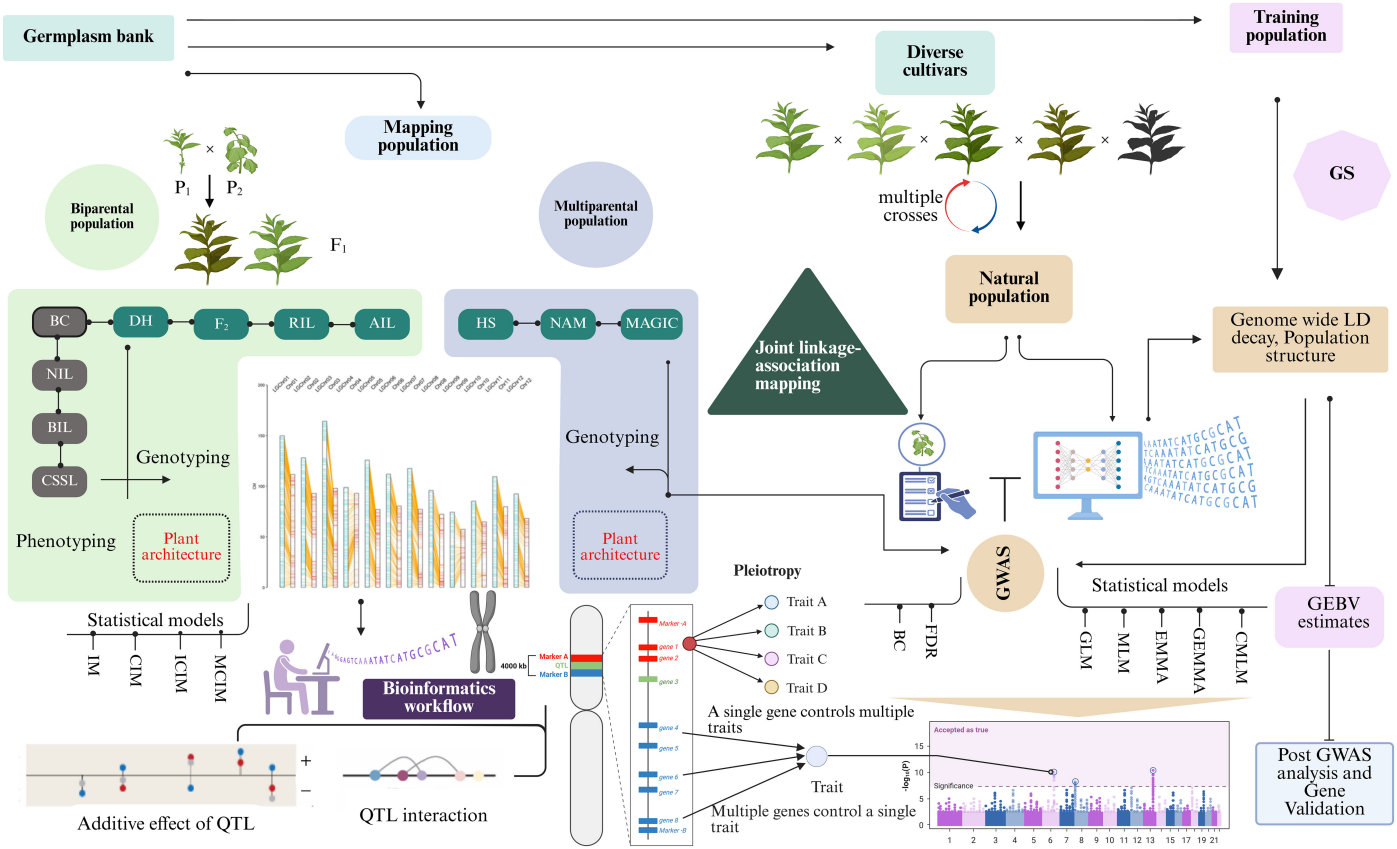
Schematic workflow for high resolution dissection of complex traits, illustrating the use of bi-parental, multi-parental and training population. Integration of MAGIC population with joint linkage-association mapping and with GEBVs enables the robust dissection of pleiotropy and epistasis and subsequent identification of candidate loci.

Despite demonstrated utility for linkage and association mapping, the analytical potential of MAGIC populations remains substantially underexploited. The future trajectory of MAGIC-enabled research encompasses three interconnected domains (**Figure 5**): high-resolution joint linkage-association mapping for causal gene identification, integration with genomic selection frameworks for accelerated breeding, and multi-omics systems biology approaches for mechanistic trait dissection. These synergistic analytical paradigms offer transformative opportunities for elucidating complex quantitative trait architectures and enhancing genetic gain in crop improvement programs.

### 10.1 High-resolution fine-mapping via Joint Linkage and Association (JLA) analysis

Achieving unprecedented fine-mapping resolution for complex QTLs through joint linkage-association (JLA) analysis represents a critical advancement for MAGIC populations. JLA frameworks synergistically combine the high statistical power of linkage mapping with the superior physical resolution of GWAS (Li et al., 2014; Wu et al., 2016; Zhuang et al., 2025) (**Figure 5**). The unique haplotype mosaic architecture of MAGIC populations (embodying mosaics of founder haplotypes extensively recombined over multiple intercrossing generations) enables JLA approaches to simultaneously model linkage effects tracking recombination events and association effects tracing specific founder allele contributions (Akinlade et al., 2025). This dual modeling addresses fundamental limitations of biparental populations and diversity panels: linkage mapping provides controlled population structure and family-relatedness correction, while association mapping exploits extensive linkage disequilibrium breakdown to achieve superior physical resolution (Würschum et al., 2012; Zhuang et al., 2025). By combining controlled genetic structure with fine-scale recombination resolution, JLA differentiates tightly linked causal variants that remain confounded in separate analyses, enabling the efficient identification of candidate genes and post-GWAS validation (Xu et al., 2024).

Despite these advantages, the potential of MAGIC populations for detecting pleiotropy and epistasis which are critical components underlying trait complexity, remains underexplored due to limited methodological development (Dwivedi et al., 2024). Advanced JLA frameworks can simultaneously analyze multiple traits using multivariate statistical modeling that quantifies founder-specific allele effects and their correlations across traits, providing robust discrimination between genuine pleiotropy (a single gene controlling multiple traits) and physical linkage of functionally independent genes (Zhou and Stephens, 2014; Bass et al., 2024). Furthermore, JLA frameworks excel at mapping epistatic interactions (where multiple genes interact to control a single trait) alongside additive main effects, capturing both synergistic and antagonistic genetic interactions (Bocianowski, 2013; Huang et al., 2015). Critically, estimating founder allele-specific epistasis reveals beneficial or deleterious allele combinations that inform optimal parental selection strategies and guide crosses that maximize transgressive segregation and genetic gain (Wang et al., 2009), elucidating genetic constraints and trade-offs shaping breeding outcomes.

### 10.2 AI and ML-enabled genomic selection in MAGIC populations for accelerated breeding

**Figure 6:**
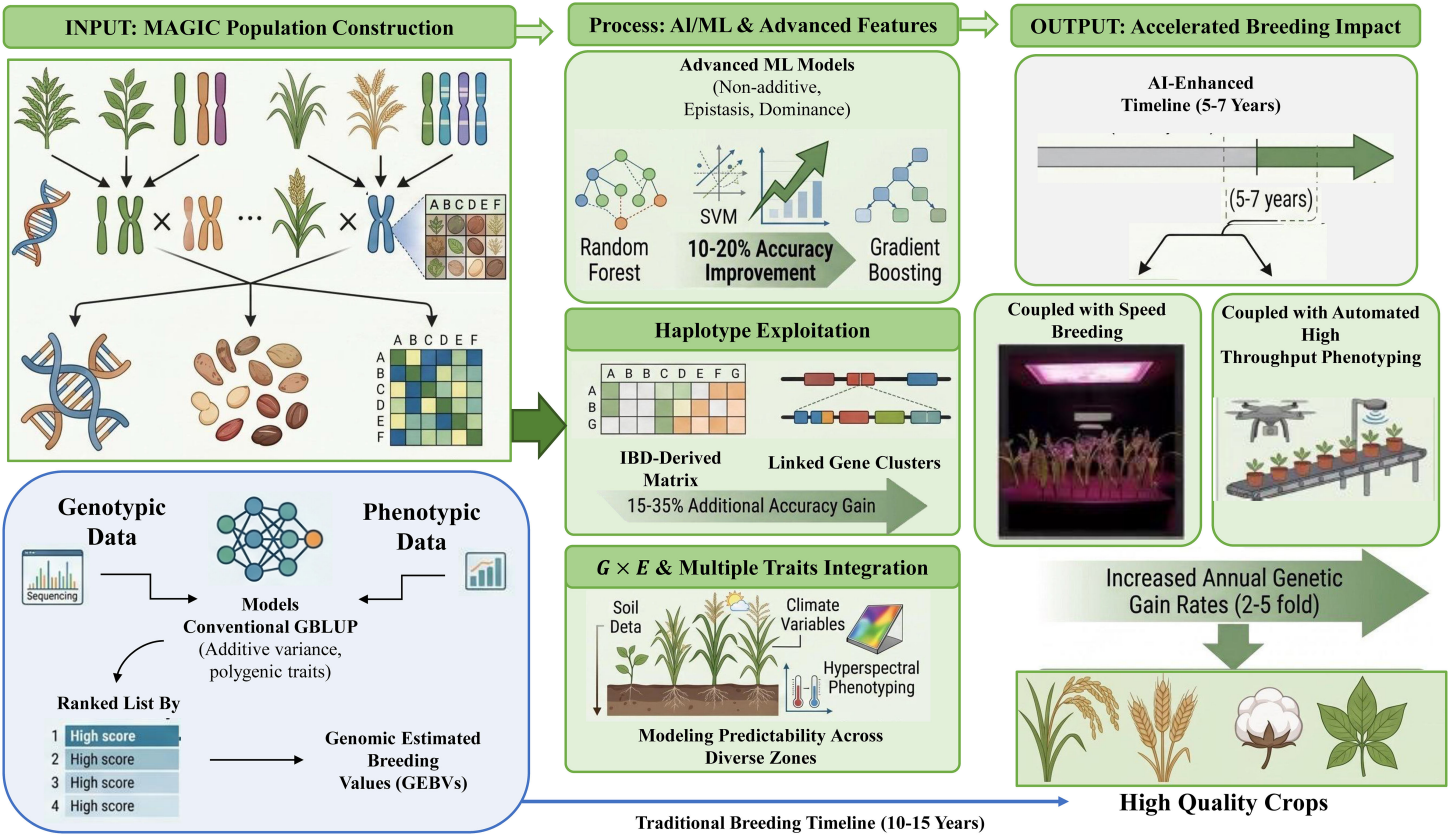
Schematic overview of MAGIC population–based breeding enhanced by machine learning, highlighting data integration, haplotype analysis, and predictive modeling. The approach demonstrates improved selection accuracy and faster development of high-quality crop varieties.

MAGIC populations have the transformative potential to redefine genomic selection by serving as optimal training sets that capture authentic QTL effects with minimal confounding (Huang et al., 2015; Arrones et al., 2020). Empirical studies demonstrate that genomic selection models trained on 8-founder MAGIC populations maintain superior prediction accuracy and transferability due to genome-wide LD decay and balanced population structure as compared to biparental or natural populations (Lorenz et al., 2011; Puglisi et al., 2021). Within the conventional frameworks, standard linear models like genomic best linear unbiased prediction (GBLUP) are routinely enhanced by incorporating identical-by-descent (IBD) derived haplotype probability matrices, which allow models to trace multi-allelic founder mosaic blocks rather than isolated biallelic markers to better capture complex polyploid architectures (Bian et al., 2021; Weber et al., 2023). While these linear and haplotype-based approaches are well-validated for capturing additive genetic variance. There is an emerging methodological shift that focuses on deploying machine learning (ML) architectures (such as random forests, support vector machines, and gradient boosting) to model non-additive genetic effects such as epistasis and dominance. These ML models are increasingly applied to complex traits where linear assumptions fail, such as secondary metabolite biosynthesis or yield stability. ML-based genomic prediction offers 10–20% accuracy improvements over conventional linear models (Kassem, 2025) (**Figure 6**). However, their empirical advantage remains highly dependent on the quality and diversity of the underlying training panel. Beyond these current ML applications, the use of deep learning networks for fully automated, multi-trait AI prediction incorporating dynamic environmental covariates (e.g., real-time weather, soil sensors, and microclimate data) remains a highly prospective, speculative direction, currently limited by the computational complexity of modeling high-dimensional genotype-by-environment interactions across shifting agro-ecological zones.

To translate these AI-driven selection models into actual genetic gain, breeding programs are increasingly pairing advanced predictive algorithms with integrated operational technologies designed to overcome the distinct biological and logistical bottlenecks inherent to large-scale multi-parent mapping. Specifically, the prolonged developmental timeline historically required to intercross multiple founders and fix highly recombined lines is now actively compressed by incorporating speed breeding protocols, which utilize controlled photoperiods and temperature regimes to fast-track generation turnover (Hickey et al., 2019). Once these massive populations are rapidly established, the subsequent bottleneck of phenotyping thousands of distinct multi-parent individuals is resolved through automated high-throughput phenotyping (HTP) platforms, providing the massive datasets required to train and refine predictive AI networks. Finally, the ultimate prospect for utilizing AI-enabled MAGIC data lies in transitioning from passive phenotypic selection of favorable recombinants to precise targeted modification via multiplexed plant genome editing. By deploying CRISPR-based technologies, breeders can directly validate and engineer the precise multi-allelic networks and linked gene clusters identified by machine learning models, bypassing traditional generational recombination altogether to rapidly deliver fixed, elite, climate-resilient cultivars (Kamran et al., 2026).

### 10.3 Advanced multi-omics integration and systems biology approaches

The integration of multi-omics profiling within Multi-parent Advanced Generation Inter-Cross (MAGIC) frameworks has established a reliable baseline for mapping intermediate molecular phenotypes, particularly through transcriptomic and metabolomic layers. Currently, expression quantitative trait loci (eQTL) and metabolite quantitative trait loci (mQTL) mapping are the most mature methods used to bridge the gap between multi-allelic genotypes and complex phenotypes (Huang et al., 2015; Arrones et al., 2020; Zhong et al., 2025). These established approaches have successfully identified key regulatory checkpoints and metabolic hubs governing traits such as stress adaptation and nutrient accumulation. However, a major limitation of current eQTL and mQTL studies in MAGIC populations fail to capture the post-translational modifications that dictate actual protein function (Alemu et al., 2025).

For this, the prospective expansion of the MAGIC framework involves the vertical integration of emerging omics layers, including proteomics, phenomics, and epigenomics. DNA methylation mapping represents a critical direction for understanding how founder-specific chromatin states influence the expression of linked gene clusters in allopolyploid crops (Ding and Chen, 2018). Furthermore, layering proteomic data onto existing transcriptomic maps will allow breeders to distinguish between transcriptional abundance and functional protein activity (Alemu et al., 2025). By synthesizing these heterogeneous data layers, breeding protocols can transition from identifying isolated markers to engineering entire optimized molecular pathways.

This systems-level approach is expected to resolve the functional redundancy often found in large-genome industrial crops, enabling breeders to stack favorable alleles across different omics levels to stabilize yield and quality across varying environmental conditions. Consequently, while transcriptomics and metabolomics provide the current foundation, the strategic addition of emerging omics layers will transform MAGIC populations into biological knowledge platforms capable of directing high-precision, climate-resilient crop improvement.

## 11 Concluding remarks

MAGIC populations represent a transformative advance in plant genetics, bridging traditional linkage mapping and association mapping while circumventing their limitations. Their unique architecture enables simultaneous multi-allelic mapping with superior resolution, comprehensive epistatic characterization, and genotype-by-environment dissection. Successful deployment across diverse crops has revealed novel genetic variation for critical breeding objectives. To fully utilize the potential of the MAGIC population, there is an immense need to optimize population size, diallel crossing schemes to break tight linkages, and automated high-throughput phenotyping, and must overcome critical bioinformatic bottlenecks, specifically the development of automated, ploidy-aware phasing pipelines for complex allopolyploids, dynamic machine learning models for genotype-by-environment interactions, and non-linear systems biology frameworks for multi-omics data integration. As agriculture confronts climate change and resource constraints, MAGIC populations provide indispensable platforms for sustainable crop improvement, enabling the transformation from empirical selection to precision-guided genetic design essential for global food security.

## Supplementary material

Supplementary Table 1 List of software used for QTL discovery across all population types, including statistical models supported.

Supplementary Table 2 Current status of MAGIC populations across all crop species (cereals, legumes, horticultural, and industrial crops).

Supplementary Figure 1 Interdependent factors affecting the precision of haplotype mosaic reconstruction in MAGIC populations.
