# Supplementary material for: MAGIC populations: a next-generation framework for dissecting complex quantitative traits and accelerating molecular breeding in crops
Source: Front Plant Sci. 2026 Jun 30;17:1867756. doi: 10.3389/fpls.2026.1867756 (PMC13364981; doi:10.3389/fpls.2026.1867756)
Supplement: Supplementary Table 1 — List of software used for QTL discovery across all population types, including statistical models supported. [file Table1.docx]

**Supplementary Table 1:** List of Software used for QTL discovery

| **Software** | **Populations Supported** | **Statistical Models** |
| --- | --- | --- |
| PLABQTL | F_2_, BC, RIL | CIM, SIM |
| QGene | F_2_, BC, RIL, DH | IM, CIM, MQM |
| MapManagerQTX | F_2_, BC, RIL | IM, Permutation, CIM |
| Windows QTL Cartographer | F_2_, BC, RIL, DH | IM, CIM, MIM |
| MCQTL | F_2_, BC, RIL | MQM |
| GMM | F_2_, BC, RIL, DH | IM, CIM |
| QTL IciMapping | F_2_, BC, RIL, DH, CSSL, NAM | ICIM, ICIM-EPI, Joint mapping across environments |
| QTLNetwork | F_2_, BC, RIL, DH | MCIM |
| R/qtl | F_2_, BC, RIL, DH | IM, CIM, MIM, Haley-Knott reg |
| GAPL | F_2_, BC, RIL, MAGIC | GLM |
| GAHP | F_2_, BC, RIL | IM, ICIM |
| fullsibQTL | Outcross full-sib families | CIM with linkage phase |
| statgenMPP | Multi-parent (incl. biparental) | Multi-parent QTL, IBD |
| R/qtl2 | F_2_, RIL, BC, DH, Multi-parent | HMM genotype probs + LMM, Association mapping |
| R/qtlbim | F_2_, BC, RIL | Bayesian IM/MIM, RJ-MCMC |
| postQTL | F_2_, RIL | Stepwise model selection + CIM |
| solQTL | F_2_ (Solanaceae) | IM |
| FlexQTL | F_2_, BC, RIL, MPP, Pedigree | Bayesian multipoint QTL, IBD estimation |
| MutMap+ | Mutant × WT F_2_, BC | SNP-index |
| QTL-seq | Bulked F_2_, BC, RIL | (SNP-index), simulation-based CI |
| QTLseqr | Bulked F_2_, BC, RIL | (SNP-index), G-method |
| MutMap | Mutant F_2_ progeny | SNP-index |
| diaQTL | MPP, Diallel populations | IM, Multiple QTL, Bayesian MIM |
| polyqtlR | Autopolyploid biparentals | IBD probabilities |
| MapMaker/QTL | F_2_, BC | IM (Max likelihood) |
| GCTA-QTL | Pedigree, GWAS panels | REML variance component mapping |
| ICIM (method) | F_2_, BC, RIL | Inclusive CIM |
| TASSEL | Natural popualtion, MPPs | GLM, MLM |
| GAPPIT | Natural popualtion, MPPs | GLM, MLM, CMLM |
| PLINK | RIL, Natural popualtion, MPPs | LMM, Population structure, allele/genotype |
| wgaim (R) | RIL, BC, DH, MAGIC | Whole-genome average interval mapping (WGAIM) |
| HAPPY | HS, MAGIC | IM |
| RABBIT | MAGIC | HMM |
| R/mpMap | MAGIC | IM, CIM, LMM |
| R/MagicQTL | MAGIC, NAM, ROAM | IM, CIM, Random model |
| mppQTL | MAGIC, NAM, ROAM | LMM |
