# Supplementary material for: MAGIC populations: a next-generation framework for dissecting complex quantitative traits and accelerating molecular breeding in crops
Source: Front Plant Sci. 2026 Jun 30;17:1867756. doi: 10.3389/fpls.2026.1867756 (PMC13364981; doi:10.3389/fpls.2026.1867756)
Supplement: Supplementary Table 2 — Current status of MAGIC populations across all crop species (cereals, legumes, horticultural, and industrial crops). [file Table2.docx]

**Supplementary Table 2**: Current status of MAGIC population in crops

| **Crop** | **Parents and Design** | **Final RILs** | **Target Traits** | **Analysis Software** | **Reference** |
| --- | --- | --- | --- | --- | --- |
| *Arabidopsis thaliana* | 19-way | 1026 | Germination date and bolting time | HAPPY | [1] |
|  | 8-way | 532 | Flowering time and leaf morphology | GenStat | [2] |
| Rice (*Oryza sativa*) | 8-way *indica,* | 1328 | Biotic/abiotic stress and grain quality | TASSEL | [3] |
|  | *8-way japonica* | 500 |  |  |  |
|  | 4-way DC1, | 271 | Agronomic traits | TASSEL | [4] |
|  | 4-way DC2 | 268 |  |  |  |
|  | 8-way | 532 |  |  |  |
|  | DC1+DC2 | 539 |  |  |  |
|  | DC1+DC2+8-way | 1071 |  |  |  |
|  | 8-way | 981 | Days to heading and culm length | rrBLUP, qqman, QTL cartographer | [5] |
|  | 4-way DC1, | 218 | Ferrous, Zinc, and Aluminum tolerance | TASSEL | [6] |
|  | 4-way DC2, | 210 |  |  |  |
|  | 8-way | 445 |  |  |  |
|  | DC12 | 428 |  |  |  |
|  | RMPRIL | 873 |  |  |  |
|  | 4-way DC1, | 271 | Heading date and plant height | TASSEL | [7] |
|  | 4-way DC2, | 268 |  |  |  |
|  | 8-way | 532 |  |  |  |
|  | 4-way | 248 | Grain shape and chalkiness | R/MagicQTL | [8] |
|  | 4-way DC1 | 215 | Zinc accumulation in grains | TASSEL | [9] |
|  | 4-way DC1 | 215 | Magnesium uptake | TASSEL | [10] |
|  | 8-way indica | 391 | Salinity and sodicity tolerance | TASSEL | [11] |
|  | 16 way | - | Seed traits under drought conditions | R/GAPIT | [12] |
|  | 16 way | 2100 | Grain yield and grain quality | R/mpMap | [13] |
|  |  |  |  | R/Asreml |  |
|  | 8-way | 372 | Grain shape | rrBLUP package | [5] |
|  | 8-way indica | 240 S4 | Bacterial disease resistance | R/mpMap | [14] |
|  |  | 340 S8 |  | TASSEL |  |
|  | 8 way | - | Agronomic and biofortification trait | TASSEL | [15] |
|  | 16-way | 18421 | 16 agronomic traits | PLINK, | [16] |
|  |  |  |  | rMVP |  |
| Wheat (*Triticum aestivum*) | 4-way | 1579 | Plant height and bectolitre weight | R/mpMap | [17] |
|  | 8-way | 1091 | Flowering time | R/mpMap | [18] |
|  | 8-way | 486 | Leaf blotch and Glume blotch | R/qtl | [19] |
|  |  | 498 |  | R/mpMap |  |
|  | 8-way | 1091 | Awning | GAPIT | [20] |
|  | 8-way | 3000 | Recombination and structural variation | R/mpMap2 | [21] |
|  |  |  |  | R/mpMapinteractive2 |  |
|  |  |  |  | magicCalling |  |
|  | 4-way | 338 | Agronomic traits | R/mpMap | [22] |
|  |  |  |  | TASSEL |  |
|  | 8-way | 394 | Powdery mildew disease resistance | R/mpMap | [23] |
|  | 8-way | 208 | Whole leaf senescence | HAPPY | [24] |
|  | 8-way | 910 | Plant height | SAS | [25] |
|  | 8-way | 1091 | Linkage map and genome diversity | R/mpMap | [26] |
|  | 4-way | - | Rhizosheath (long root hairs) | MPWGAIM | [27] |
|  | 8-way |  |  |  |  |
|  | 8-way | ~1000 | Plant area mm2, plant height, senescence, water amount | HAPPY | [28] |
|  | 16-way | 596 | Agronomic traits | HMM, HaplotypeCaller, | [29] |
|  |  |  |  | PLINK |  |
|  | 8-way | 394 | Leaf rust resistance | R/mpMap | [30] |
|  | 4-way | 1458 | Coleoptile and shoot growth | WGAIM | [31] |
| Barley (*Hordeum vulgare*) | 8-way | 122, 29, 81, 303 | Powdery mildew | GAPIT | [32] |
|  | 8-way | 352 | Grain yield | GP and RKHS regression models | [33] |
|  | 8-way | 5000 | Flowering time, heading date | R/mpmap, SAS | [34] |
|  | Diverse panel | 6160 | Flowering time, agronomy | Custom R/scripts, PLINK | [35] |
|  | 8-way | 533 | Flowering time | PLINK | [36] |
|  | 32-way | 324 | Plant genetic diversity and evolutionary research | - | [37] |
| Maize (*Zea mays*) | 8-way | 672 | Resistance to Fusarium Ear RotÊ | SAS | [38] |
|  | 8-way | 672 | Leaf senescence physiological traits | SAS, TASSEL | [39] |
|  | 8-way | 529 | Population diversity, Grain yield | SAS, R/qtl, QTLRel | [40] |
|  | 8-way | 672 | Resistance to corn borers, agronomic traits | SAS, TASSEL | [41] |
|  | 16-way | 550 | Agronomic and yield related traits | R/qtl2, R/GridLMM | [42] |
|  | 4-way | 1291 | Agronomic traits | SAS | [43] |
|  | 4-way | 708 | Agronomic and yield related traits | rMVP, mashr, | [44] |
|  | 4-way | 708 | qualitative traits (seed and plant color, awns) and drought tolerance | R/qtl2,TASSEL | [45] |
|  | 19-way | 1000 | yield and drought tolerance | TASSEL | [46] |
| Chickpea (*Cicer arietinum*) | 8-way | 1135 | Flowering time and plant height | GAPIT | [47] |
|  | 8-way | 1136 | Agronomic traits, drought and heat tolerance | - | [48] |
| Common bean (*Phaseolus vulgaris*) | 8-way | 996 | Agronomic traits | TASSEL, GAPIT | [49] |
|  | 4-way | 1050 | Resistance to white mold | R/qtl2, custom pipeline for GWAS | [50] |
| Soybean (*Glycine max*) | 8-way | 721 | Agronomy and seed quality traits | TASSEL, GAPL | [51] |
| Cowpea (*Vigna unguiculata*) | 8-way | 305 | Agronomic and yield related traits and drought stress index | BLINK, rrBLUP | [52] |
|  | 8-way | 305 | Flowring time and seed size | TASSEL, rrBLUP | [53] |
|  | 8-way | 291 | Nutrient assays | R/mpmap, BGLR | [54] |
|  | 8-way | 305 | Biotic and abiotic stress, seed quality and agronomic traits | R/mpmap | [54] |
| Fababean (*Vicia faba)* | 11-way | 189 | Frost tolerance | TASSEL | [55] |
|  |  |  |  |  |  |
|  | 7-way | 2678 accessions | Agronomic traits | PLINK, GAPIT | [56] |
|  | 4-way | 1000 | Morphological and biochemical traits | - | [57] |
| Tomato (*Solanum lycopersicum*) | 8-way | 397 | Fuit Weight | R/mpmap, TASSEL | [58] |
|  | 8-way | 354 | fruit size, leaf morphology and plant pigmentation | TASSEL, GAPIT | [59] |
|  | 8-way | 400 | resistance to fungi, bacteria and viruses | - | [60] |
| Tobacco (*Nicotiana tabacum*) | 8-way | 800 | Nicotine content | TASSEL | [61] |
|  | 8-way | <800 | Agronomic traits | QTL Ici mapping, TASSEL | [62] |
|  | 8-way | <800 | Leaf width plasticity | GCTA, rrBLUP | [63] |
| Mustard (*Brassica juncea*) | 8-way | 408 | glucosinolate traits | TASSEL | [64] |
| Rapeseed (*Brassica napus*) | 8-way | 680 | Agronomic and yield related traits | - | [65] |
| Sunflower (*Helianthus annuus*) | 11-way | ~2000 | plant architecture, disease resistance, oil content and quality | - | [66, 67] |
| Lettuce (*Lactuca sativa*) | 16-way | 381 | Agronomic traits | GEMMA | [67] |
| Cotton (*Gossypium hirsutum*) | 11-way | 550 | Fiber quality traits | PLINK, FAST-LMM | [68] |
|  | 16-way | 920 | lint yield and fiber quality | PLINK, EMMAX | [69] |
|  | 16-way | 372 | Fiber quality traits | GAPIT | [70] |
|  | 11-way | 550 | Fiber length trait | GAPIT | [71] |
|  | 11-way | 550 | Fiber quality | GAPIT, TASSEL | [72] |
|  | 21-way | 180 | population diversity and Mosiac IBD origins | TASSEL | [73] |
|  | 8-way | 320 | Verticillium wilt resistance | TASSEL | [74] |
|  | 12-way | 1500 | Fibre yield and quality | - | [75] |

**References**

1. Kover PX, Valdar W, Trakalo J, Scarcelli N, Ehrenreich IM, Purugganan MD, et al. A multiparent advanced generation inter-cross to fine-map quantitative traits in Arabidopsis thaliana. PLoS Genet. 2009;5. https://doi.org/10.1371/journal.pgen.1000551.

2. Huang X, Paulo M-J, Boer M, Effgen S, Keizer P, Koornneef M, et al. Analysis of natural allelic variation in Arabidopsis using a multiparent recombinant inbred line population. Proceedings of the National Academy of Sciences. 2011;108:4488–93. https://doi.org/10.1073/pnas.1100465108.

3. Bandillo N, Raghavan C, Muyco PA, Sevilla MAL, Lobina IT, Dilla-Ermita CJ, et al. Multi-parent advanced generation inter-cross (MAGIC) populations in rice: Progress and potential for genetics research and breeding. Rice. 2013;6. https://doi.org/10.1186/1939-8433-6-11.

4. Meng L, Zhao X, Ponce K, Ye G, Leung H. QTL mapping for agronomic traits using multi-parent advanced generation inter-cross (MAGIC) populations derived from diverse elite indica rice lines. Field Crops Res. 2016;189:19–42. https://doi.org/10.1016/j.fcr.2016.02.004.

5. Ogawa D, Nonoue Y, Tsunematsu H, Kanno N, Yamamoto T, Yonemaru J. Discovery of QTL Alleles for Grain Shape in the Japan-MAGIC Rice Population Using Haplotype Information. G3 Genes|Genomes|Genetics. 2018;8:3559–65. https://doi.org/10.1534/g3.118.200558.

6. Meng L, Wang B, Zhao X, Ponce K, Qian Q, Ye G. Association mapping of ferrous, zinc, and aluminum tolerance at the seedling stage in Indica rice using MAGIC populations. Front Plant Sci. 2017;8. https://doi.org/10.3389/fpls.2017.01822.

7. Meng L, Guo L, Ponce K, Zhao X, Ye G. Characterization of Three Indica Rice Multiparent Advanced Generation Intercross (MAGIC) Populations for Quantitative Trait Loci Identification. Plant Genome. 2016;9:plantgenome2015.10.0109. https://doi.org/https://doi.org/10.3835/plantgenome2015.10.0109.

8. Ayaad M, Han Z, Zheng K, Hu G, Abo-Yousef M, Sobeih SElS, et al. Bin-based genome-wide association studies reveal superior alleles for improvement of appearance quality using a 4-way MAGIC population in rice. J Adv Res. 2021;28:183–94. https://doi.org/https://doi.org/10.1016/j.jare.2020.08.001.

9. Liu S, Zou W, Lu X, Bian J, He H, Chen J, et al. Genome-wide association study using a multiparent advanced generation intercross (Magic) population identified qtls and candidate genes to predict shoot and grain zinc contents in rice. Agriculture (Switzerland). 2021;11:1–14. https://doi.org/10.3390/agriculture11010070.

10. Zhi S, Zou W, Li J, Meng L, Liu J, Chen J, et al. Mapping QTLs and gene validation studies for Mg2+ uptake and translocation using a MAGIC population in rice. Front Plant Sci. 2023;14. https://doi.org/10.3389/fpls.2023.1131064.

11. Krishnamurthy SL, Sharma PC, Dewan D, Lokeshkumar BM, Rathor S, Warraich AS, et al. Genome wide association study of MAGIC population reveals a novel QTL for salinity and sodicity tolerance in rice. Physiology and Molecular Biology of Plants. 2022;28:819–35. https://doi.org/10.1007/s12298-022-01174-8.

12. Marrano A, Moyers BT. Scanning the rice Global MAGIC population for dynamic genetic control of seed traits under vegetative drought. The Plant Phenome Journal. 2022;5:e20033. https://doi.org/https://doi.org/10.1002/ppj2.20033.

13. Zaw H, Raghavan C, Pocsedio A, Swamy BPM, Jubay ML, Singh RK, et al. Exploring genetic architecture of grain yield and quality traits in a 16-way indica by japonica rice MAGIC global population. Sci Rep. 2019;9. https://doi.org/10.1038/s41598-019-55357-7.

14. Bossa-Castro AM, Tekete C, Raghavan C, Delorean EE, Dereeper A, Dagno K, et al. Allelic variation for broad-spectrum resistance and susceptibility to bacterial pathogens identified in a rice MAGIC population. Plant Biotechnol J. 2018;16:1559–68. https://doi.org/https://doi.org/10.1111/pbi.12895.

15. Descalsota GIL, Swamy BPM, Zaw H, Inabangan-Asilo MA, Amparado A, Mauleon R, et al. Genome-wide association mapping in a rice magic plus population detects qtls and genes useful for biofortification. Front Plant Sci. 2018;9. https://doi.org/10.3389/fpls.2018.01347.

16. Wei X, Chen M, Zhang Q, Gong J, Liu J, Yong K, et al. Genomic investigation of 18,421 lines reveals the genetic architecture of rice. Science (1979). 2024;385. https://doi.org/10.1126/science.adm8762.

17. Huang BE, George AW, Forrest KL, Kilian A, Hayden MJ, Morell MK, et al. A multiparent advanced generation inter-cross population for genetic analysis in wheat. Plant Biotechnol J. 2012;10:826–39. https://doi.org/10.1111/j.1467-7652.2012.00702.x.

18. Fourquet L, Barber T, Campos-Mantello C, Howell P, Orman-Ligeza B, Percival-Alwyn L, et al. An eight-founder wheat MAGIC population allows fine-mapping of flowering time loci and provides novel insights into the genetic control of flowering time. Theoretical and Applied Genetics. 2024;137:277. https://doi.org/10.1007/s00122-024-04787-7.

19. Lin M, Corsi B, Ficke A, Tan K-C, Cockram J, Lillemo M. Genetic mapping using a wheat multi-founder population reveals a locus on chromosome 2A controlling resistance to both leaf and glume blotch caused by the necrotrophic fungal pathogen Parastagonospora nodorum. Theoretical and Applied Genetics. 2020;133:785–808. https://doi.org/10.1007/s00122-019-03507-w.

20. Mackay IJ, Bansept-Basler P, Barber T, Bentley AR, Cockram J, Gosman N, et al. An Eight-Parent Multiparent Advanced Generation Inter-Cross Population for Winter-Sown Wheat: Creation, Properties, and Validation. G3 Genes|Genomes|Genetics. 2014;4:1603–10. https://doi.org/10.1534/g3.114.012963.

21. Shah R, Huang BE, Whan A, Fradgley NS, Newberry M, Verbyla K, et al. Recombination and structural variation in a large 8-founder wheat MAGIC population. G3 Genes|Genomes|Genetics. 2025;15:jkaf037. https://doi.org/10.1093/g3journal/jkaf037.

22. Milner SG, Maccaferri M, Huang BE, Mantovani P, Massi A, Frascaroli E, et al. A multiparental cross population for mapping QTL for agronomic traits in durum wheat (Triticum turgidum ssp. durum). Plant Biotechnol J. 2016;14:735–48. https://doi.org/https://doi.org/10.1111/pbi.12424.

23. Stadlmeier M, Hartl L, Mohler V. Usefulness of a multiparent advanced generation intercross population with a greatly reduced mating design for genetic studies in winter wheat. Front Plant Sci. 2018;871. https://doi.org/10.3389/fpls.2018.01825.

24. Camargo A V., Mott R, Gardner KA, Mackay IJ, Corke F, Doonan JH, et al. Determining phenological patterns associated with the onset of senescence in a wheat magic mapping population. Front Plant Sci. 2016;7. https://doi.org/10.3389/fpls.2016.01540.

25. Sannemann W, Lisker A, Maurer A, Léon J, Kazman E, Cöster H, et al. Adaptive selection of founder segments and epistatic control of plant height in the MAGIC winter wheat population WM-800. BMC Genomics. 2018;19:559. https://doi.org/10.1186/s12864-018-4915-3.

26. Gardner KA, Wittern LM, Mackay IJ. A highly recombined, high‐density, eight‐founder wheat MAGIC map reveals extensive segregation distortion and genomic locations of introgression segments. Plant Biotechnol J. 2016;14:1406–17.

27. Delhaize E, Rathjen TM, Cavanagh CR. The genetics of rhizosheath size in a multiparent mapping population of wheat. J Exp Bot. 2015;66:4527–36. https://doi.org/10.1093/jxb/erv223.

28. Camargo A V., Mackay I, Mott R, Han J, Doonan JH, Askew K, et al. Functional mapping of quantitative trait loci (QTLS) associated with plant performance in a wheat MAGIC mapping population. Front Plant Sci. 2018;9. https://doi.org/10.3389/fpls.2018.00887.

29. Scott MF, Fradgley N, Bentley AR, Brabbs T, Corke F, Gardner KA, et al. Limited haplotype diversity underlies polygenic trait architecture across 70 years of wheat breeding. Genome Biol. 2021;22:137. https://doi.org/10.1186/s13059-021-02354-7.

30. Rollar S, Serfling A, Geyer M, Hartl L, Mohler V, Ordon F. QTL mapping of adult plant and seedling resistance to leaf rust (Puccinia triticina Eriks.) in a multiparent advanced generation intercross (MAGIC) wheat population. Theoretical and Applied Genetics. 2021;134:37–51. https://doi.org/10.1007/s00122-020-03657-2.

31. Rebetzke GJ, Verbyla AP, Verbyla KL, Morell MK, Cavanagh CR. Use of a large multiparent wheat mapping population in genomic dissection of coleoptile and seedling growth. Plant Biotechnol J. 2014;12:219–30. https://doi.org/https://doi.org/10.1111/pbi.12130.

32. Novakazi F, Krusell L, Jensen JD, Orabi J, Jahoor A, Bengtsson T. You had me at “magic”!: Four barley magic populations reveal novel resistance qtl for powdery mildew. Genes (Basel). 2020;11:1–21. https://doi.org/10.3390/genes11121512.

33. Puglisi D, Delbono S, Visioni A, Ozkan H, Kara İ, Casas AM, et al. Genomic Prediction of Grain Yield in a Barley MAGIC Population Modeling Genotype per Environment Interaction. Front Plant Sci. 2021;12. https://doi.org/10.3389/fpls.2021.664148.

34. Sannemann W, Huang BE, Mathew B, Léon J. Multi-parent advanced generation inter-cross in barley: high-resolution quantitative trait locus mapping for flowering time as a proof of concept. Molecular Breeding. 2015;35:86. https://doi.org/10.1007/s11032-015-0284-7.

35. Hemshrot A, Poets AM, Tyagi P, Lei L, Carter CK, Hirsch CN, et al. Development of a multiparent population for genetic mapping and allele discovery in six-row barley. Genetics. 2019;213:595–613. https://doi.org/10.1534/genetics.119.302046.

36. Mathew B, Léon J, Sannemann W, Sillanpää MJ. Detection of Epistasis for Flowering Time Using Bayesian Multilocus Estimation in a Barley MAGIC Population. Genetics. 2018;208:525–36. https://doi.org/10.1534/genetics.117.300546.

37. Bülow L, Nachtigall M, Frese L. A MAGIC population as an approach to the conservation and development of genetic diversity of winter barley for breeding purposes by on-farm management. Journal fur Kulturpflanzen. 2019;71:286–98. https://doi.org/10.5073/JfK.2019.11.02.

38. Butrón A, Santiago R, Cao A, Samayoa LF, Malvar RA. QTLs for Resistance to Fusarium Ear Rot in a Multiparent Advanced Generation Intercross (MAGIC) Maize Population. Plant Dis. 2018;103:897–904. https://doi.org/10.1094/PDIS-09-18-1669-RE.

39. Caicedo M, Munaiz ED, Malvar RA, Jiménez JC, Ordas B. Precision Mapping of a Maize MAGIC Population Identified a Candidate Gene for the Senescence-Associated Physiological Traits. Front Genet. 2021;12. https://doi.org/10.3389/fgene.2021.716821.

40. Dell’Acqua M, Gatti DM, Pea G, Cattonaro F, Coppens F, Magris G, et al. Genetic properties of the MAGIC maize population: a new platform for high definition QTL mapping in Zea mays. Genome Biol. 2015;16:167.

41. Jiménez-Galindo JC, Malvar RA, Butrón A, Santiago R, Samayoa LF, Caicedo M, et al. Mapping of resistance to corn borers in a MAGIC population of maize. BMC Plant Biol. 2019;19:431. https://doi.org/10.1186/s12870-019-2052-z.

42. Odell SG, Hudson AI, Praud S, Dubreuil P, Tixier MH, Ross-Ibarra J, et al. Modeling allelic diversity of multiparent mapping populations affects detection of quantitative trait loci. G3: Genes, Genomes, Genetics. 2022;12. https://doi.org/10.1093/g3journal/jkac011.

43. Mahan AL, Murray SC, Klein PE. Four-Parent Maize (FPM) Population: Development and Phenotypic Characterization. Crop Sci. 2018;58:1106–17. https://doi.org/https://doi.org/10.2135/cropsci2017.07.0450.

44. Kumar N, Boatwright JL, Brenton ZW, Sapkota S, Ballén-Taborda C, Myers MT, et al. Development and characterization of a sorghum multiparent advanced generation intercross (MAGIC) population for capturing diversity among seed parent gene pool. G3: Genes, Genomes, Genetics. 2023;13. https://doi.org/10.1093/g3journal/jkad037.

45. Kumar N, Boatwright JL, Sapkota S, Brenton ZW, Ballén-Taborda C, Myers MT, et al. Discovering useful genetic variation in the seed parent gene pool for sorghum improvement. Front Genet. 2023;14. https://doi.org/10.3389/fgene.2023.1221148.

46. Ongom PO, Ejeta G. mating design and genetic structure of a multi-parent advanced generation intercross (MAGIC) population of sorghum (Sorghum bicolor (L.) moench). G3: Genes, Genomes, Genetics. 2018;8:331–41. https://doi.org/10.1534/g3.117.300248.

47. Akinlade OJ, Robinson H, Kang Y, Thudi M, Samineni S, Gaur P, et al. A chickpea MAGIC population to dissect the genetics of complex traits. Plant Genome. 2025;18. https://doi.org/10.1002/tpg2.70096.

48. Samineni S, Sajja SB, Mondal B, Chand U, Thudi M, Varshney RK, et al. MAGIC lines in chickpea: Development and exploitation of genetic diversity. Euphytica. 2021;217:137.

49. Diaz S, Ariza-Suarez D, Izquierdo P, Lobaton JD, de la Hoz JF, Acevedo F, et al. Genetic mapping for agronomic traits in a MAGIC population of common bean (Phaseolus vulgaris L.) under drought conditions. BMC Genomics. 2020;21. https://doi.org/10.1186/s12864-020-07213-6.

50. Escobar E, Oladzad A, Simons K, Miklas P, Lee RK, Schroder S, et al. New genomic regions associated with white mold resistance in dry bean using a MAGIC population. Plant Genome. 2022;15. https://doi.org/10.1002/tpg2.20190.

51. Hashemi SM, Perry G, Rajcan I, Eskandari M. SoyMAGIC: An Unprecedented Platform for Genetic Studies and Breeding Activities in Soybean. Front Plant Sci. 2022;13. https://doi.org/10.3389/fpls.2022.945471.

52. Ravelombola W, Shi A, Huynh B-L. Loci discovery, network-guided approach, and genomic prediction for drought tolerance index in a multi-parent advanced generation intercross (MAGIC) cowpea population. Hortic Res. 2021;8:24. https://doi.org/10.1038/s41438-021-00462-w.

53. Olatoye MO, Hu Z, Aikpokpodion PO. Epistasis detection and modeling for genomic selection in cowpea (Vigna unguiculata. L. Walp.). Frontiers in Genetics. 2019;10 JUN. https://doi.org/10.3389/fgene.2019.00677.

54. Huynh B-L, Stangoulis JCR, Vuong TD, Shi H, Nguyen HT, Duong T, et al. Quantitative trait loci and genomic prediction for grain sugar and mineral concentrations of cowpea [Vigna unguiculata (L.) Walp.]. Sci Rep. 2024;14:4567. https://doi.org/10.1038/s41598-024-55214-2.

55. Sallam A, Martsch R. Association mapping for frost tolerance using multi-parent advanced generation inter-cross (MAGIC) population in faba bean (Vicia faba L.). Genetica. 2015;143:501–14. https://doi.org/10.1007/s10709-015-9848-z.

56. Skovbjerg CK, Angra D, Robertson-Shersby-Harvie T, Kreplak J, Keeble-Gagnère G, Kaur S, et al. Genetic analysis of global faba bean diversity, agronomic traits and selection signatures. Theoretical and Applied Genetics. 2023;136. https://doi.org/10.1007/s00122-023-04360-8.

57. Khazaei H, Stoddard FL, Purves RW, Vandenberg A. A multi-parent faba bean (Vicia faba L.) population for future genomic studies. Plant Genetic Resources. 2018;16:419–23.

58. Pascual L, Desplat N, Huang BE, Desgroux A, Bruguier L, Bouchet JP, et al. Potential of a tomato MAGIC population to decipher the genetic control of quantitative traits and detect causal variants in the resequencing era. Plant Biotechnol J. 2015;13:565–77. https://doi.org/10.1111/pbi.12282.

59. Arrones A, Antar O, Pereira-Dias L, Solana A, Ferrante P, Aprea G, et al. A novel tomato interspecific (Solanum lycopersicum var. cerasiforme and Solanum pimpinellifolium) MAGIC population facilitates trait association and candidate gene discovery in untapped exotic germplasm. Hortic Res. 2024;11:uhae154. https://doi.org/10.1093/hr/uhae154.

60. Campanelli G, Sestili S, Acciarri N, Montemurro F, Palma D, Leteo F, et al. Multi-parental advances generation inter-cross population, to develop organic tomato genotypes by participatory plant breeding. Agronomy. 2019;9. https://doi.org/10.3390/agronomy9030119.

61. Yuan G, Sun K, Yu W, Jiang Z, Jiang C, Liu D, et al. Development of a MAGIC population and high-resolution quantitative trait mapping for nicotine content in tobacco. Front Plant Sci. 2023;13. https://doi.org/10.3389/fpls.2022.1086950.

62. Liu Y, Yuan G, Si H, Sun Y, Jiang Z, Liu D, et al. Identification of QTLs Associated With Agronomic Traits in Tobacco via a Biparental Population and an Eight-Way MAGIC Population. Front Plant Sci. 2022;13. https://doi.org/10.3389/fpls.2022.878267.

63. Liu L, Liu W, Sun Y, Han Y, Hao R, Zhang W, et al. Complex genetic architecture underlying the plasticity of tobacco leaf width provides insight into across-environment genomic prediction. 2024. https://doi.org/10.1101/2024.05.05.592603.

64. Yan W, Zhao H, Yu K, Wang T, Khattak AN, Tian E. Development of a multiparent advanced generation intercross (MAGIC) population for genetic exploitation of complex traits in Brassica juncea: Glucosinolate content as an example. Plant Breeding. 2020;139:779–89. https://doi.org/10.1111/pbr.12820.

65. Fu-yong Z, Heng Z, Xiao-ling W, Xiao-fang LI. Construction and application potential of MAGIC population on genetic breeding of rapeseed (Brassica napus L.). Chinese Journal of Oil Crop Sciences. 2017;39.

66. Domínguez M, Colombo D, Filippi C, Ben Guerrero E, Dillcheneider A, Corro Molas A, et al. Building MAGIC populations: Novel resources for sunflower trait improvement. Oil Crop Science. 2025;10:131–43. https://doi.org/10.1016/j.ocsci.2025.05.001.

67. Chen H, Chen J, Zhai R, Lavelle D, Jia Y, Tang Q, et al. Dissecting the genetic architecture of key agronomic traits in lettuce using a MAGIC population. Genome Biol. 2025;26. https://doi.org/10.1186/s13059-025-03541-6.

68. Wang M, Qi Z, Thyssen GN, Naoumkina M, Jenkins JN, McCarty JC, et al. Genomic interrogation of a MAGIC population highlights genetic factors controlling fiber quality traits in cotton. Commun Biol. 2022;5. https://doi.org/10.1038/s42003-022-03022-7.

69. Li Y, Si Z, Wang G, Shi Z, Chen J, Qi G, et al. Genomic insights into the genetic basis of cotton breeding in China. Mol Plant. 2023;16:662–77. https://doi.org/10.1016/j.molp.2023.01.012.

70. Mohammed J, Thyssen GN, Hinze L, Zhang J, Zeng L, Fang DD. A GWAS identified loci and candidate genes associated with fiber quality traits in a new cotton MAGIC population. Theoretical and Applied Genetics. 2024;138:10. https://doi.org/10.1007/s00122-024-04800-z.

71. Naoumkina M, Thyssen GN, Fang DD, Jenkins JN, McCarty JC, Florane CB. Genetic and transcriptomic dissection of the fiber length trait from a cotton (Gossypium hirsutum L.) MAGIC population. BMC Genomics. 2019;20:112. https://doi.org/10.1186/s12864-019-5427-5.

72. Islam MS, Thyssen GN, Jenkins JN, Zeng L, Delhom CD, McCarty JC, et al. A MAGIC population-based genome-wide association study reveals functional association of GhRBB1_A07 gene with superior fiber quality in cotton. BMC Genomics. 2016;17. https://doi.org/10.1186/s12864-016-3249-2.

73. Fang DD, Thyssen GN, Wang M, Jenkins JN, McCarty JC, Jones DC. Genomic confirmation of Gossypium barbadense introgression into G. hirsutum and a subsequent MAGIC population. Molecular Genetics and Genomics. 2023;298:143–52. https://doi.org/10.1007/s00438-022-01974-3.

74. Ayyaz M, Chang Z, Ding S, Han P, Xu L, Abudukeyoumu A, et al. QTL mapping associated with Verticillium wilt resistance in cotton based on MAGIC population. Journal of Cotton Research. 2025;8:9. https://doi.org/10.1186/s42397-025-00211-7.

75. Li DG, Li ZX, Hu JS, Lin ZX, Li XF. Polymorphism analysis of multi-parent advanced generation inter-cross (MAGIC) populations of upland cotton developed in China. Genet Mol Res. 2016;15:10–4238.
