# Supplementary material for: MAGIC populations: a next-generation framework for dissecting complex quantitative traits and accelerating molecular breeding in crops
Source: Front Plant Sci. 2026 Jun 30;17:1867756. doi: 10.3389/fpls.2026.1867756 (PMC13364981; doi:10.3389/fpls.2026.1867756)
Supplement: Supplementary Figure 1 — Interdependent factors affecting the precision of haplotype mosaic reconstruction in MAGIC populations. [file Image1.pdf]

Supplementary Figure 1

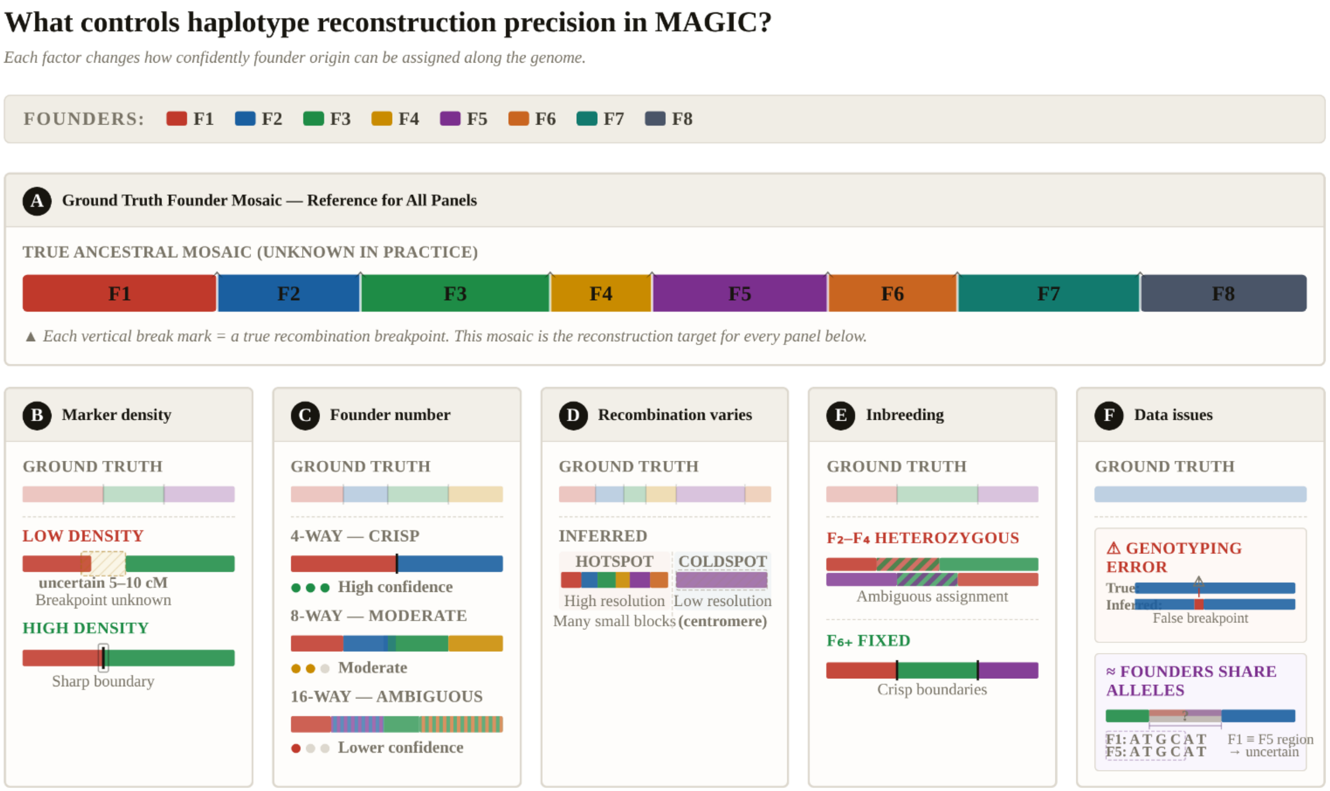

The true ancestral mosaic of a MAGIC RIL — a sequence of founder-colored blocks separated by true recombination breakpoints (marked by ▲)This is the reconstruction target; it is unknown in practice. In panels **B–F**, the faded top track reproduces the ground truth for direct comparison; the solid bottom track shows the inferred mosaic under each condition. **(B)** Sparse markers leave wide breakpoint uncertainty intervals (hatched band); dense markers allow sub-cM localization. **(C)** As founder number increases (4 → 8 → 16-way), assignment confidence drops; mixed-color patches indicate regions where the correct founder cannot be resolved. Dot scores (●●●) summarise relative confidence. **(D)** Recombination hotspots produce many short, precisely resolved blocks; pericentromeric coldspots yield large, uncertain regions (hatched overlay). **(E)** Early-generation lines (F_2–F_4) carry residual heterozygosity (striped diploid tracks), blurring founder boundaries; advanced RILs (F_{6+}) are fully fixed, enabling crisp inference. **(F)** Two data artifacts: isolated genotyping errors create false single-marker crossovers (⚠ spike); regions of identical alleles between founders (F1 ≡ F5) produce grey ambiguous patches resolved only by flanking informative loci.
